# Root plasticity and xylem modifications drive drought resilience in okra [*Abelmoschus esculentus* (L.) Moench] at the seedling stage

**DOI:** 10.3389/fpls.2025.1630935

**Published:** 2025-11-19

**Authors:** Sukhjiwan Jeet Kaur, Nilesh Talekar, Priyanka Upadhyay, Shailesh Kumar Singh, Monika Kumari, Dinesh Kumar Saini, Bal Krishna, Shivani Lalotra, Habiburahman Ayoubi

**Affiliations:** 1 Department of Genetics and Plant Breeding, School of Agriculture, Lovely Professional University, Phagwara, Punjab, India; 2 Department of Plant Breeding and Genetics, Dr. Rajendra Prasad Central Agricultural University, Samastipur, Bihar, India; 3 Department of Horticulture, School of Agriculture, Lovely Professional University, Phagwara, Punjab, India; 4 Department of Molecular Biology and Genetic Engineering, Lovely Professional University, Phagwara, Punjab, India; 5 Department of Plant Breeding and Genetics, Punjab Agricultural University, Ludhiana, Punjab, India; 6 Department of Genetics and Plant Breeding, Bihar Agricultural University, Sabour, Bhagalpur, Bihar, India; 7 Department of Agronomy, School of Agriculture, Lovely Professional University, Phagwara, Punjab, India

**Keywords:** okra, drought, root plasticity, physio-biochemical responses, xylem architecture

## Abstract

Okra [*Abelmoschus esculentus* (L.) Moench] plays a vital role in ensuring food and nutritional security in arid and semi-arid regions; however, its growth is severely limited by drought stress. While root plasticity and physio-biochemical responses are known to contribute to drought resilience, their specific roles in okra remain underexplored. This study assessed drought tolerance in 55 okra genotypes subjected to three levels of PEG 6000-induced osmotic stress (0%, 10%, and 20%) under polyhouse conditions. Drought stress delayed germination and significantly reduced key growth parameters, including leaf number, shoot length, fresh and dry biomass, and survival rate. Root traits such as secondary root number, root length, and fresh root weight also declined, although the root-to-shoot ratio increased under severe stress, indicating an adaptive shift in biomass allocation. Biochemical analyses revealed elevated levels of chlorophyll, carotenoids, and proline in response to drought, reflecting enhanced stress tolerance mechanisms. Based on overall performance, genotypes G51 (Sonam), G6 (HAU-480), G10 (Bhindi Champion), and G45 (Pooja-01) emerged as the most drought-tolerant, exhibiting superior root development and biomass accumulation. Oxidative stress markers, MDA and H_2_O_2_, also increased significantly under severe drought, further validating physiological damage and supporting the classification of tolerant and susceptible genotypes. Principal component analysis identified the mean productivity index and tolerance index as key contributors to genotypic variation under stress. Additionally, Field Emission Scanning Electron Microscopy (FESEM) revealed genotype-specific xylem adaptations, with reduced vessel size in drought-tolerant genotypes likely mitigating the risk of embolism. These findings highlight the importance of root plasticity, xylem architecture, and biochemical adjustments in conferring drought tolerance in okra. Prioritizing traits such as secondary root formation and reduced xylem vessel size offers promising avenues for breeding resilient okra cultivars suited to water-limited environments.

## 1 Introduction

Abiotic stresses such as temperature, water deficit, radiation, and nutrient imbalance account for approximately 51-82% of global crop yield losses (Oshunsanya et al., 2019). Among these, drought is a major constraint that reduces both water availability and quality, thereby threatening sustainable agricultural productivity. According to the World Resources Institute, about 25% of global crops are cultivated in regions where water supply is either highly stressed, highly unreliable, or both (World Resources Institute, 2024). Factors such as accelerated urbanization, rapid population growth, and climate change are projected to intensify water scarcity across more than 80% of global croplands by 2050, with vegetable crops being particularly vulnerable (Liu et al., 2022). Vegetables, due to their succulent nature and high-water content (approximately 90%), are highly sensitive to drought stress, which adversely affects their growth, development, and yield (Abbas et al., 2023). This reduction in vegetable production contributes to the escalation of the global hunger index, malnutrition, and increasing mortality rates (Mansoor et al., 2022). Addressing these challenges requires the identification and development of drought-tolerant crop varieties with adaptive root anatomy and physio-biochemical responses under water-limited conditions.

Plant responses to water deficit depend on the duration of stress, its rate of imposition, and the developmental stage during which it occurs. Germination and early seedling growth are particularly affected, resulting in reduced seedling vigor, root and shoot length, and fresh and dry biomass (Gao et al., 2023). Drought also disrupts key cellular and physiological processes, ultimately impairing overall plant development (Khanna, 2024). Drought adaptation is genotype-dependent and involves complex mechanisms such as osmotic adjustment, stomatal regulation, antioxidant defense, and root system modifications (Bray, 2001; Seleiman et al., 2021). Under drought conditions, plants optimize resource use by altering growth patterns, reducing total biomass, and reallocating resources to roots for improved water uptake (Wang et al., 2024; Osakabe et al., 2014). To conserve water, stomatal conductance is reduced, which limits CO_2_ uptake and leads to decreased photosynthetic pigment levels (Wang et al., 2018). In parallel, drought triggers oxidative stress marked by the accumulation of reactive oxygen species (ROS), particularly malondialdehyde (MDA) and hydrogen peroxide (H_2_O_2_), both of which serve as reliable biochemical indicators of cellular damage (Noctor et al., 2014). MDA, a byproduct of lipid peroxidation, reflects membrane integrity loss and increases with stress intensity (Khaleghi et al., 2019). H_2_O_2_, while indicative of oxidative stress, also acts as a critical signalling molecule that activates downstream defense responses, including the accumulation of carotenoids and proline, key components that contribute to oxidative stress tolerance and osmotic adjustment (Zareyan et al., 2025; Gowtham et al., 2022; Rao et al., 2025).

Roots play a central role in drought tolerance, being the first organs to sense water stress. Significant variability in root morphology is observed among plant species and genotypes within species (Contreras-Soto et al., 2022). To enhance water transport under limited conditions, plants adjust xylem vessel anatomy by reducing vessel diameter or increasing vessel density (Kong et al., 2023). Smaller vessel diameters improve hydraulic safety and minimize the risk of embolism (Li et al., 2024), while increased vessel numbers facilitate efficient water transport from roots to shoots. These xylem-mediated drought responses are genotype-specific, with each variety exhibiting distinct structural and physiological adaptations (Kettani et al., 2024).

Okra (*Abelmoschus esculentus* L. Moench) is a nutritionally valuable vegetable crop widely grown in tropical and subtropical regions. It is rich in dietary fiber, minerals, vitamins, antioxidants, and proteins essential for human health (Ibitoye and Kolawole, 2022). Despite its relative drought tolerance, yield losses of 30-100% have been reported under water-limited conditions (Mkhabela et al., 2023). Drought significantly reduces okra seedling growth traits such as plant height, stem diameter, leaf area, and biomass, as well as root traits including root fresh and dry weight (Torun et al., 2023). To mitigate water loss and oxidative stress, drought-tolerant okra genotypes exhibit enhanced physiological and biochemical responses such as efficient stomatal closure, greater proline accumulation, reduced transpiration, and elevated antioxidant activity (Razi and Muneer, 2023). Additionally, okra roots adapt structurally by increasing root length, surface area, and volume to enhance water and nutrient uptake in response to drought intensity (Mahmood et al., 2024). While general physiological responses have been studied, limited attention has been given to the anatomical traits, particularly root system plasticity and xylem vessel architecture, that underlie drought adaptation in okra. This study addresses this critical gap by evaluating 55 diverse genotypes for key seedling-stage responses under osmotic stress, integrating root morphological plasticity, xylem vessel modifications, and physio-biochemical traits. The findings aim to identify robust, early-stage indicators of drought resilience, offering a comprehensiveapproach for breeding drought-tolerant okra cultivars.

## 2 Materials and methods

The current study evaluated the drought tolerance of 55 okra genotypes (**Supplementary Table 1**) at the seedling stage under three different water regimes. For comparing the drought responses of the genotypes, a check variety Pusa Sawani (G54) was included in the study. The experiment was conducted under controlled polyhouse conditions using a completely randomized design (CRD) at Lovely Professional University, Phagwara, Punjab, India (31.2450°N latitude; 75.7010°E longitude). The polyhouse environment was maintained at a constant temperature of 25°C and 50% relative humidity. Seeds of all 55 genotypes were soaked overnight in water before being sown in 250 mL virgin plastic pots filled with a uniform mixture of cocopeat, vermicompost, and perlite. Six seeds per genotype per pot per treatment were sown in three replications. Upon seedling emergence (seven-eight days after sowing), only one seedling per cup was retained to ensure proper seedling growth.

Drought stress was induced using Polyethylene Glycol (PEG 6000) solutions at three levels: T0 (0%, control), T1 (10%), and T2 (20%). The PEG solutions were prepared by dissolving 100 g and 200 g of PEG 6000 in 1 L of distilled water for the T1 and T2 treatments, respectively, while the control (T0) contained only distilled water. Drought stress was imposed by applying 15 mL of the respective PEG solution to each container every 48 hours, starting from the date of sowing. Among the 55 genotypes, 37 successfully germinated, while 18 failed to sprout due to reduced seed viability. All observations were recorded 25 days after the seedling emergence.

### 2.1 Evaluation of growth, root, biochemical, and anatomical traits under three PEG 6000 regimes

#### 2.1.1 Growth and root traits

Growth parameters were recorded as follows: days to seed germination (DSG) was calculated as the number of days from sowing to seedling emergence. The number of leaves (NOL) and secondary roots (NSR) were manually counted at the end of the experiment. Shoot length (SL, cm) and root length (RL, cm) were measured using a scale, while root fresh weight (RFW, g) and total fresh weight (TFW, g) were recorded using a weighing balance (ATOM Selves- MH 200). Total dry weight (TDW, g) was determined after oven-drying fresh samples at 80°C until a constant weight was achieved. The root-to-shoot ratio (R/S) was calculated by dividing root length by shoot length. The survival rate (SR) was determined as the percentage of plants that survived in each treatment. Abbreviations for all estimated parameters, along with their classifications, trait descriptions, and units, are provided in **Supplementary Table 2**.

#### 2.1.2 Biochemical traits

All biochemical parameters were recorded 25 days after seedling emergence. Chlorophyll and carotenoid contents were estimated following the method outlined by Arnon (1949). Fresh leaf samples (0.25 g) were ground in 80% acetone, and the mixture was then centrifuged at 3000 rpm for 15 minutes. The absorbance of the supernatant was recorded at 663, 647, and 470 nm using a visible spectrophotometer 168. The concentrations of chlorophyll and carotenoids were calculated using the formulas provided by Ayub et al. (2021). Further, proline content was measured following the method of Bates et al. (1973). A 0.50 g leaf sample was mixed with 3% sulfosalicylic acid and centrifuged at 3000 rpm for 15 minutes. After centrifugation, 200 µL of the supernatant was taken and mixed with 200 µL of glacial acetic acid and 200 µL of ninhydrin. The mixture was kept in a water bath at 100°C for 1 hour, and the reaction was stopped by placing it in an ice bath. After cooling, 400 µL of toluene was added, and the upper layer was extracted. Its absorbance was then measured at 520 nm.


$$Chla\;\left(mg/g\right)\;=\;\left(12.25\times A_{663.2}–\;2.79\times A_{646.8}\right)$$

$$Chlb\;\left(mg/g\right)=\;\left(21.21\times A_{646.8}\;–\;5.1\times A_{663.2}\right)$$

ChlT (mg/g)= Chla + Chlb

$$Carotenoids\;\left(µg/mL\right)\;=\;\left(1000A_{470}-1.82Chla-85.02Chlb\right)/\;198$$

##### 2.1.2.1 Assessment of lipid peroxidation and hydrogen peroxide

To assess oxidative damage under drought stress, lipid peroxidation and hydrogen peroxide (H_2_O_2_) accumulation were quantified through MDA and H_2_O_2_ measurements. These analyses were conducted only under T0 (control) and T2 (severe stress) treatments to capture the contrast between non-stressed and highly stressed conditions, thereby maximizing the physiological resolution of drought-induced oxidative responses. Including the intermediate treatment (T1) was deemed unnecessary for this analysis, as prior studies have shown that the most pronounced oxidative effects typically occur at maximum stress levels, while minimal or no effects are evident under control conditions (Pravisya et al., 2019).

Leaf tissue (0.20 g) was homogenized in 5 mL of ice-cold 0.1% (w/v) trichloroacetic acid (TCA). The homogenate was then centrifuged at 12,000 rpm for 15 minutes, and the supernatant was collected for the quantification of MDA and H_2_O_2_. The MDA content was determined following the protocol of Verma and Dubey (2003). Briefly, 0.5 mL of the supernatant was mixed with 1 mL of 20% (w/v) TCA containing 0.5% (w/v) thiobarbituric acid (TBA). The mixture was incubated at 95°C for 30 minutes, and the reaction was immediately terminated in an ice bath. After incubation, the samples were centrifuged at 14,000 rpm for 15 minutes, and the absorbance of the supernatant was measured at 532 and 600 nm using a Visible Spectrophotometer 168. H_2_O_2_ concentration was measured according to the method described by Velikova et al. (2000). For the assay, 500 µL of the supernatant was mixed with 500 µL of 10 mM potassium phosphate buffer (pH 7.0) and 1 mL of 1 M potassium iodide (KI). The reaction mixture was incubated in the dark at room temperature for 20 minutes, and the absorbance was recorded at 390 nm using the same spectrophotometer.

#### 2.1.3 Field emission scanning electron microscopy analysis

Fresh root samples obtained from 25-day-old plants were gradually dehydrated through a graded ethanol series (70%, 90%, and 100%), with each step lasting 15 minutes, following the method described by Talbot and White (2013). After dehydration, the roots were cut into cross sections and coated with a thin layer of gold using a JEOL Smart Coater. The root structure of the samples was then analyzed using a Field Emission Scanning Electron Microscope (FE-SEM) (JEOL, Japan, with OXFORD EDS, LN2-free). The samples were observed at magnifications of 600x for primary root xylem vessel size (PRXVS) and at 1500x for secondary root xylem vessel size (SRXVS) under both control and drought stress conditions.

### 2.2 Statistical analysis

Descriptive statistics and Pearson’s correlation coefficients (r) for the estimated traits were calculated using Minitab^®^ 21.4 statistical software (Minitab LLC, State College, PA, United States) and R statistical software (version 4.2.2, R Core Team, 2022) in RStudio (version 2022.07.2 build 576, RStudio Team, 2022), respectively. Analysis of variance (ANOVA) based on a generalized linear model was performed using Minitab^®^ 21.4 statistical software to evaluate the significant effects of genotype, treatment (PEG 6000 level), replication, and the genotype × treatment interaction for all measured traits. Drought tolerance classification was performed using two approaches: First, following the methods of Osborne and Rengel (2002) and Aziz et al. (2011), the okra genotypes were classified as efficient (E), medium (M), and inefficient (I) based on the absolute values assigned to each genotype. These values were determined using the population mean (µ) and standard deviation (SD) of each parameter under both controlled and water-deficit conditions. The mean value for efficient genotypes was > µ + SD, for medium genotypes it ranged between µ + SD and µ - SD, and for inefficient genotypes it was < µ - SD. The scores assigned to efficient, medium, and inefficient genotypes were 3, 2, and 1, respectively. The distinct scores for each parameter were summed to obtain the cumulative score for each genotype (Reddy et al., 2021). Second, drought tolerance indices were calculated based on dry weight for all genotypes, using the equations provided by Negarestani et al. (2019) and Grzesiak et al. (2019):


Stress susceptibility index (SSI) = (1−T/ C)/ (1−xT/ xC)

Mean productivity index (MPI) = (C+T)/ 2

Geometric mean productivity index (GMPI) = √C×T

Harmonic mean index (HMI) = 2(C×T)/ (C+T)

$$Stress\;tolerance\;index\;\left(STI\right)\;=\;\left(C\times T\right)/\;(xC{)}_{2}$$

Tolerance index (TI) = C−T

Stressindex (SI) = T/ C

Where C and T represent the dry weight (DW) of genotypes under control and treatment conditions, respectively, and *x*C and *x*T represent the average total seedling dry weight (DW) of all the studied genotypes under control and treatment conditions. Finally, the standardized values of all indices were used to calculate the stress tolerance score (STS) of all genotypes grown under control and drought conditions, following the equation from Thiry et al. (2016) and Negarestani et al. (2019):

Stress tolerance score (STS) = SSI + MPI + GMPI + HMI + STI + TI + SI.

## 3 Results

### 3.1 Trait-specific responses of okra genotypes under three PEG 6000 levels

**Figure 1:**
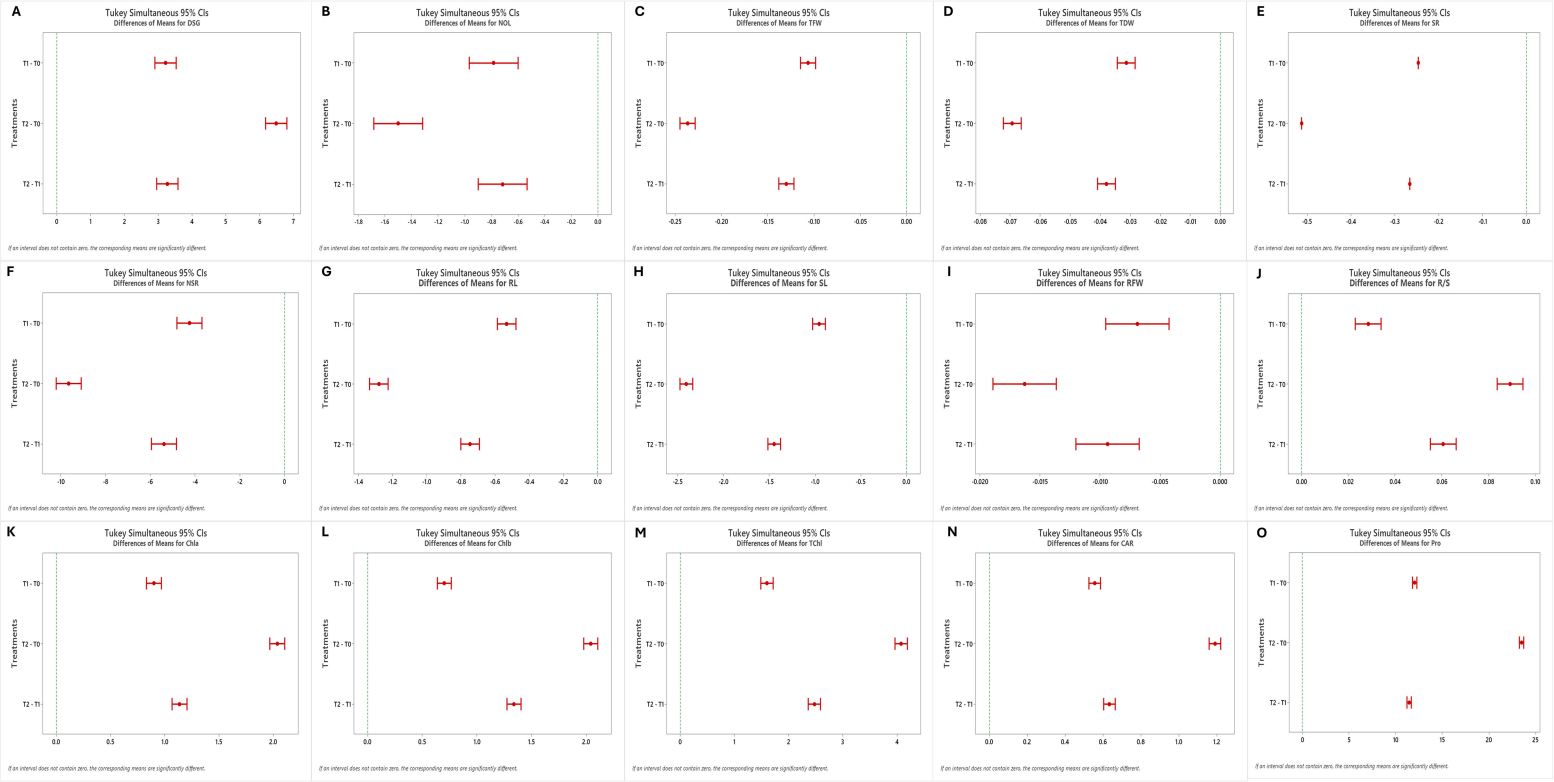
Tukey’s Pairwise Comparisons for the effects of three varying PEG 6000 concentrations as control (T0, without PEG), 10% PEG-induced stress (T1), and 20% PEG-induced stress (T2) on growth, root and biochemical parameters in okra: **(A)** Days to seed germination (DSG), **(B)** Number of leaves (NOL), **(C)** Total Fresh weight (TFW), **(D)** Total Dry weight (TDW), **(E)** Survival rate (SR), **(F)** Number of secondary roots (NSR), **(G)** Root length (RL), **(H)** Shoot length (SL), **(I)** Root fresh weight (RFW), **(J)** Root-to-shoot-ratio (R/S), **(K)** Chlorophyll a (Chla), **(L)** Chlorophyll b (Chlb), **(M)** Total Chlorophyll (TChl), **(N)** Carotenoid (CAR), **(O)** Proline (Pro).

**Figure 2:**
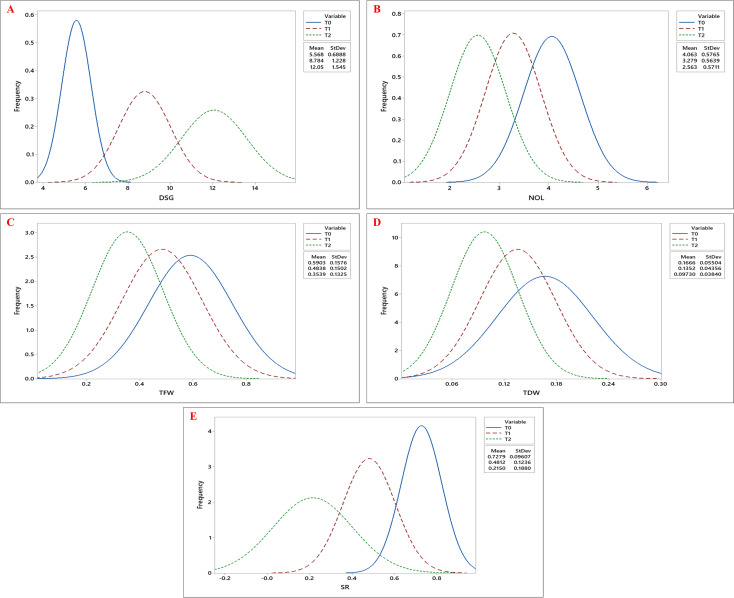
Frequency distribution curves of **(A)** Days to Seed Germination (DSG), **(B)** Number of Leaves (NOL), **(C)** Total Fresh Weight (TFW), **(D)** Total Dry Weight (TDW), and **(E)** Survival Rate (SR) across okra genotypes under three drought stress treatments: control (T0), 10% PEG 6000 (T1), and 20% PEG 6000 (T2).The three curves- blue, red, and green, represent the trait distributions under T0, T1, and T2 treatments, respectively, with their corresponding mean and standard deviation values indicated in the legend.

**Figure 3:**
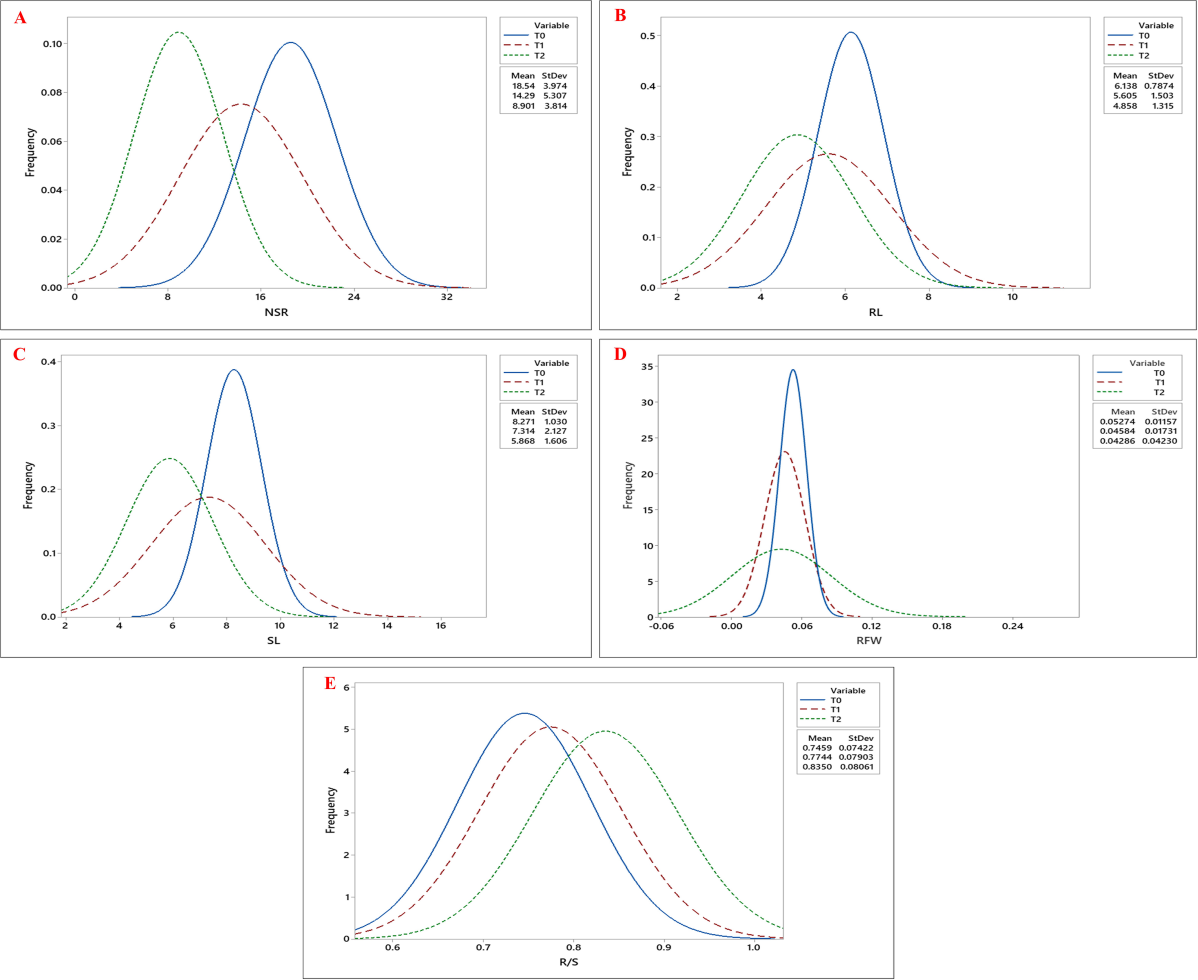
Frequency distribution curves of **(A)** Number of Secondary Roots (NSR), **(B)** Root Length (RL), **(C)** Shoot Length (SL), **(D)** Root Fresh Weight (RFW), and **(E)** Root-to-Shoot Ratio (R/S) across okra genotypes under three drought stress treatments: control (T0), 10% PEG 6000 (T1), and 20% PEG 6000 (T2). The three curves- blue, red, and green, represent the trait distributions under T0, T1, and T2 treatments, respectively, with their corresponding mean and standard deviation values indicated in the legend.

The effects of genotypes, treatments, and their interaction were highly significant (p< 0.001) for all the estimated growth, root, and biochemical parameters (**Tables 1**, **2**). Tukey’s pairwise comparison test revealed significant differences between the treatment levels (T0, T1, and T2; **Figure 1**). The mean DSG increased from 5.56 days under T0 to 8.78 and 12.05 days under T1 and T2, respectively, indicating delayed germination with increasing drought stress (**Figure 2A**; **Table 3**). Growth traits such as NOL, TFW, TDW, and SR were higher under control conditions (T0) compared to drought treatments (T1 and T2), demonstrating that vegetative growth was significantly impaired by drought stress (**Figures 2B–E**). Among the root traits, the mean values of NSR, RL, SL, and RFW progressively declined with increasing drought intensity (T0 to T2), whereas the R/S ratio increased from 0.75 at T0 to 0.77 at T1 and 0.83 at T2 (**Figures 3A–E**; **Table 3**).

**Table 1:** Analysis of variance for 15 estimated growth, root and biochemical parameters under three varying polyethylene glycol (PEG) 6000 concentrations in okra.

Variables		Genotypes	Treatments	Replication	Genotypes x Treatments	Error	Total
Mean square	DF	36	2	2	72	220	332
	DSG	8.14***	1167.59***	2.63	2.48***	0.99	
	NOL	1.94***	62.47***	0.49	0.49***	0.3342	
	TFW	0.154***	1.555***	0.00136	0.020***	0.00067	
	TDW	0.015***	0.133***	0.000058	0.002***	0.000088	
	SR	0.124	7.306	0	0.028	0	
	NSR	147.08***	2590.52***	6.6	14.21***	3.1	
	RL	8.97***	45.90***	0.0804	2.42***	0.0293	
	SL	17.15***	162.52***	0.123	3.66***	0.048	
	RFW	0.003***	0.003***	0.000714	0.002***	0.000443	
	R/S	0.042***	0.23***	0.000198	0.006***	0.000302	
	Chla	16.99***	115.47***	0.033	4.05***	0.047	
	Chlb	17.68***	119.18***	0.008	4.52***	0.041	
	TChl	54.69***	467.83***	0.009	14.19***	0.128	
	CAR	5.86***	39.29***	0.023	1.205***	0.0092	
	Pro	147.7***	15305.6***	0.6	74.5***	0.5	

***Significance at P< 0.001.

DSG, Days to seed germination; NOL, Number of leaves; TFW, Total fresh weight; TDW, Total dry weight; SR, Survival rate; NSR, Number of secondary roots; RL, Root length; SL, Shoot length; RFW, Root fresh weight; R/S, Root-to-shoot ratio; Chla, Chlorophyll a; Chlb, Chlorophyll b; TChl, Total Chlorophyll; CAR, Carotenoid; Pro, Proline.

**Table 2:** Analysis of variance for malondialdehyde (MDA) and hydrogen peroxide (H_2_O_2_) content in okra genotypes under control (T0) and drought stress (20% PEG 6000, T2) conditions.

Variables	DF	Mean Square	
		MDA	H2O2
Genotypes	36	9.11***	99.3***
Treatments	1	2332.07***	22647.1***
Replication	2	0.01	0
Genotypes x Treatments	36	4.97***	68.8***
Error	146	0.01	0
Total	221		

***Significance at P < 0.001

MDA, Malondialdehyde; H_2_O_2_, Hydrogen peroxide.

**Table 3:** Descriptive statistics for 17 estimated growth, root and biochemical parameters under three varying polyethylene glycol (PEG) 6000 concentrations in okra.

Trait	Treatment	Mean	CV	Minimum	Maximum
DSG	T0	5.56 ± 1.067	19.17	4	9
	T1	8.78 ± 1.467	16.70	5	14
	T2	12.05 ± 1.736	14.41	7	16
NOL	T0	4.06 ± 0.754	18.56	3	7
	T1	3.28 ± 0.740	22.59	2	5
	T2	2.56 ± 0.720	28.10	1	5
TFW	T0	0.59 ± 0.157	26.61	0.29	0.88
	T1	0.48 ± 0.149	30.88	0.07	0.78
	T2	0.35 ± 0.135	38.05	0.05	0.59
TDW	T0	0.17 ± 0.055	33.11	0.06	0.27
	T1	0.14 ± 0.043	32.23	0.02	0.2
	T2	0.09 ± 0.038	40.07	0.01	0.17
SR	T0	0.72 ± 0.095	13.08	0.53	0.93
	T1	0.48 ± 0.122	25.45	0.27	0.87
	T2	0.21 ± 0.186	86.65	0.07	0.8
NSR	T0	18.54 ± 4.278	23.08	9	32
	T1	14.29 ± 5.419	37.93	4	35
	T2	8.90 ± 4.011	45.07	4	24
RL	T0	6.14 ± 0.793	12.93	4.4	8.1
	T1	5.60 ± 1.495	26.68	3.9	11.2
	T2	4.86 ± 1.310	26.99	2.7	9.2
SL	T0	8.27 ± 1.039	12.57	6	4.4
	T1	7.31 ± 2.114	28.91	4.7	12.1
	T2	5.87 ± 1.601	27.29	3.4	7
RFW	T0	0.053 ± 0.012	22.06	0.018	0.077
	T1	0.046 ± 0.017	37.53	0.014	0.096
	T2	0.043 ± 0.051	120.10	0.01	0.4
R/S	T0	0.75 ± 0.074	9.98	0.58	0.88
	T1	0.77 ± 0.079	10.21	0.61	0.92
	T2	0.83 ± 0.082	9.83	0.67	0.98
Chla	T0	4.24 ± 1.646	38.83	1.309	8.81
	T1	5.14 ± 1.422	27.67	2.124	8.024
	T2	6.28 ± 1.890	30.13	2.632	9.45
Chlb	T0	3.09 ± 1.803	58.23	0.44	7.71
	T1	3.79 ± 1.461	38.45	1.14	7.73
	T2	5.14 ± 1.854	36.1	1.55	7.95
TChl	T0	7.34 ± 3.084	42.03	1.75	15.24
	T1	8.94 ± 2.419	27.07	3.58	13
	T2	11.41 ± 3.475	30.45	4.18	16.99
CAR	T0	1.94 ± 0.959	49.59	0.18	3.99
	T1	2.49 ± 0.798	32.04	0.49	3.69
	T2	3.12 ± 1.080	34.57	0.14	4.96
Pro	T0	5.61 ± 0.611	10.89	4.52	7.21
	T1	17.65 ± 4.542	25.74	9.08	27.97
	T2	29.09 ± 8.784	30.2	12.19	50.03
MDA	T0	2.07 ± 0.571	27.56	1.10	3.34
	T2	8.55 ± 2.070	24.20	5.60	13.68
H_2_O_2_	T0	12.09 ± 1.55	12.88	9.67	15.74
	T2	32.29 ± 7.25	22.46	19.89	46.52

DSG, Days to seed germination; NOL, Number of leaves; SL, Shoot length; TFW, Total fresh weight; TDW, Total dry weight; SR, Survival rate; NSR, Number of secondary roots; RL, Root length; RFW, Root fresh weight; R/S, Root-to-shoot ratio; Chla, Chlorophyll a; Chlb, Chlorophyll b; TChl, Total Chlorophyll; CAR, Carotenoid; Pro, Proline; MDA, Malondialdehyde; H_2_O_2_, Hydrogen peroxide.

**Figure 4:**
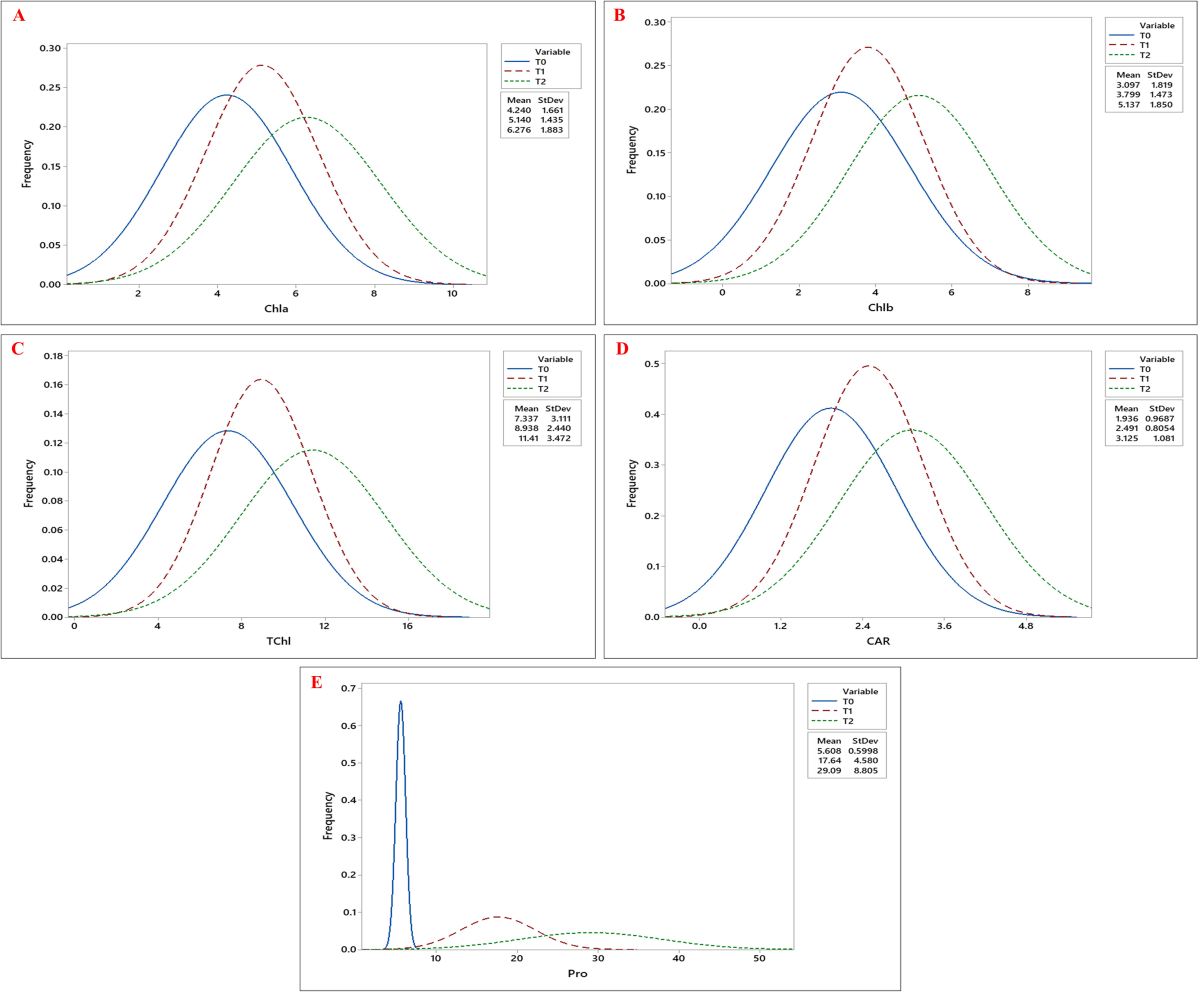
Frequency distribution curves of **(A)** Chlorophyll a (Chla), **(B)** Chlorophyll b (Chlb), **(C)** Total Chlorophyll (TChl), **(D)** Carotenoid (CAR), and **(E)** Proline (Pro) content across okra genotypes under three drought stress treatments: control (T0), 10% PEG 6000 (T1), and 20% PEG 6000 (T2).The three curves- blue, red, and green, represent the trait distributions under T0, T1, and T2 treatments, respectively, with their corresponding mean and standard deviation values indicated in the legend.

**Figure 5:**
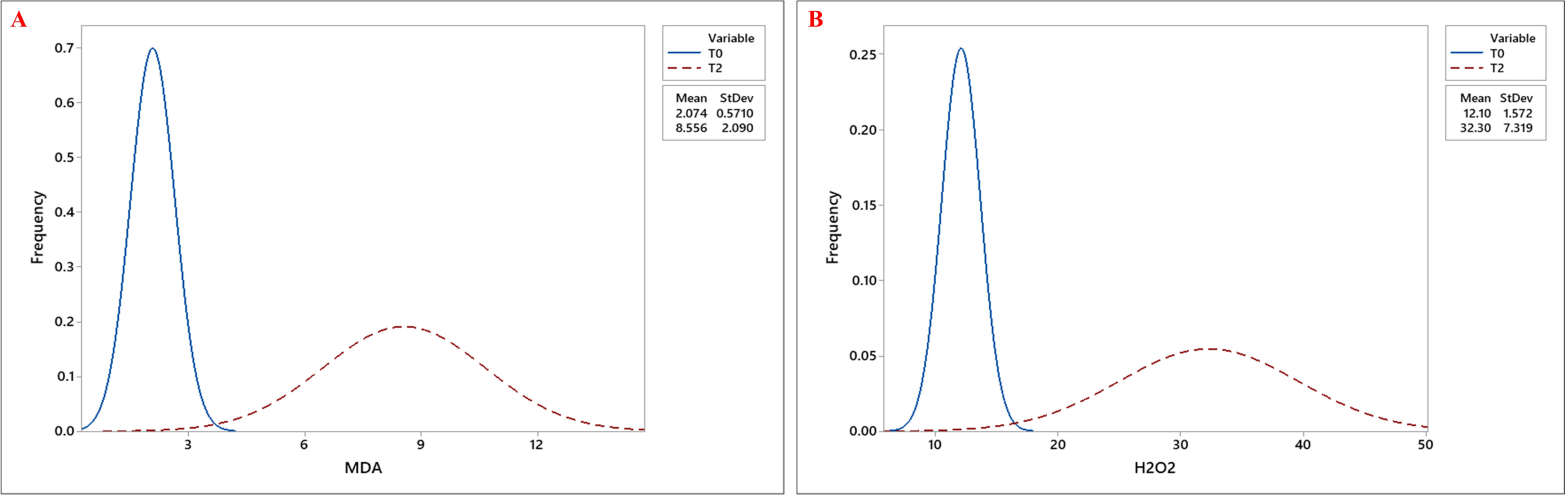
Frequency distribution curves of **(A)** Malondialdehyde (MDA) content and **(B)** Hydrogen Peroxide (H_2_O_2_) content across okra genotypes under control (T0) and severe drought stress (T2) treatments. The two curves- blue, and red, represent the trait distributions under T0, and T2 treatments, respectively, with their corresponding mean and standard deviation values indicated in the legend.

Biochemical traits, including chlorophyll a (Chla), chlorophyll b (Chlb), total chlorophyll (Tchl), and carotenoid (CAR) content, increased under drought stress, with the highest values observed at T2, followed by T1, compared to T0. Similarly, proline (Pro) accumulation was highest under T2, followed by T1, with the lowest levels observed under T0 (**Figures 4A–E**). Besides, both MDA and H_2_O_2_ levels showed significant accumulation under drought stress, with the highest mean values observed at T2 (**Figures 5A, B**; **Table 3**).

#### 3.1.1 The effects of PEG-induced drought stress on seedling growth traits

Drought stress significantly increased the number of days to seed germination while reducing other growth parameters across all genotypes (**Supplementary Figures 1A–J**). With increasing PEG concentration from T0 to T2, DSG increased by 116.5%. Despite this, genotype G51 exhibited faster germination compared to other genotypes. All growth parameters, namely, NOL, TFW, and TDW, showed significant reductions of 36.92%, 40.04%, and 41.59%, respectively, from T0 to T2. Among all genotypes, G51 and G45 demonstrated better growth performance by maintaining the highest NOL and TFW under severe drought conditions (T2). The survival rate declined sharply by 70.47% as PEG concentration increased from T0 to T2; however, genotypes G51, followed by G54 and G45, maintained relatively higher survival rates under T2.

#### 3.1.2 The effects of PEG-induced drought stress on root traits

Root morphological traits were significantly affected by drought stress, with a consistent decline observed across all genotypes (**Supplementary Figures 2A–J**). NSR decreased by 51.99% from T0 to T2, although genotypes G51 and G45 maintained comparatively higher NSR under drought conditions. RL, SL, and RFW were also reduced by 20.85%, 29.06%, and 18.72%, respectively. Despite these reductions, genotypes G51, G45, and G54 sustained relatively better RL, SL, and RFW under stress, whereas genotypes G19, G22, and G47 exhibited the most severe declines. Drought stress led to a shift in biomass allocation, favoring root growth over shoot growth to enhance water uptake from deeper soil layers. In our study, R/S increased by 11.94% from T0 to T2. This adaptive response was most evident in genotypes G49 and G54 under T2 and T1 conditions, respectively.

#### 3.1.3 The effects of PEG-induced drought stress on biochemical traits

Osmotic stress induced at the early developmental stage significantly elevated biochemical parameters such as chlorophyll and carotenoid contents across okra genotypes (**Supplementary Figures 3A–H**). Chla, Chlb, and TChl increased by 47.99%, 65.85%, and 55.53%, respectively, from T0 to T2. Among the genotypes, G9 and G22 recorded the highest Chla and TChl levels, while Chlb was highest in G6 and G44. Additionally, CAR content increased markedly by 61.42%, with genotypes G13 and G19 exhibiting the greatest accumulation. Furthermore, in response to drought stress, proline content rose sharply by 418.72% from control (T0) to severe stress (T2), with G46 and G48 showing the most pronounced response (**Supplementary Figures 3I, J**). Similarly, drought stress significantly elevated oxidative stress markers across okra genotypes, with MDA and H_2_O_2_ contents increased by 313.04% and 167.09%, respectively, from T0 to T2 (**Supplementary Figures 4A–D**). The highest accumulation of MDA and H_2_O_2_ was observed in G19, whereas genotypes G6 and G45 maintained the lowest levels under drought conditions (**Figure 5**). Individual genotypic variations for all estimated traits are presented in **Supplementary Figures 1**–**4**.

### 3.2 Correlation pattern among estimated parameters under three varying PEG 6000 regimes

**Figure 6:**
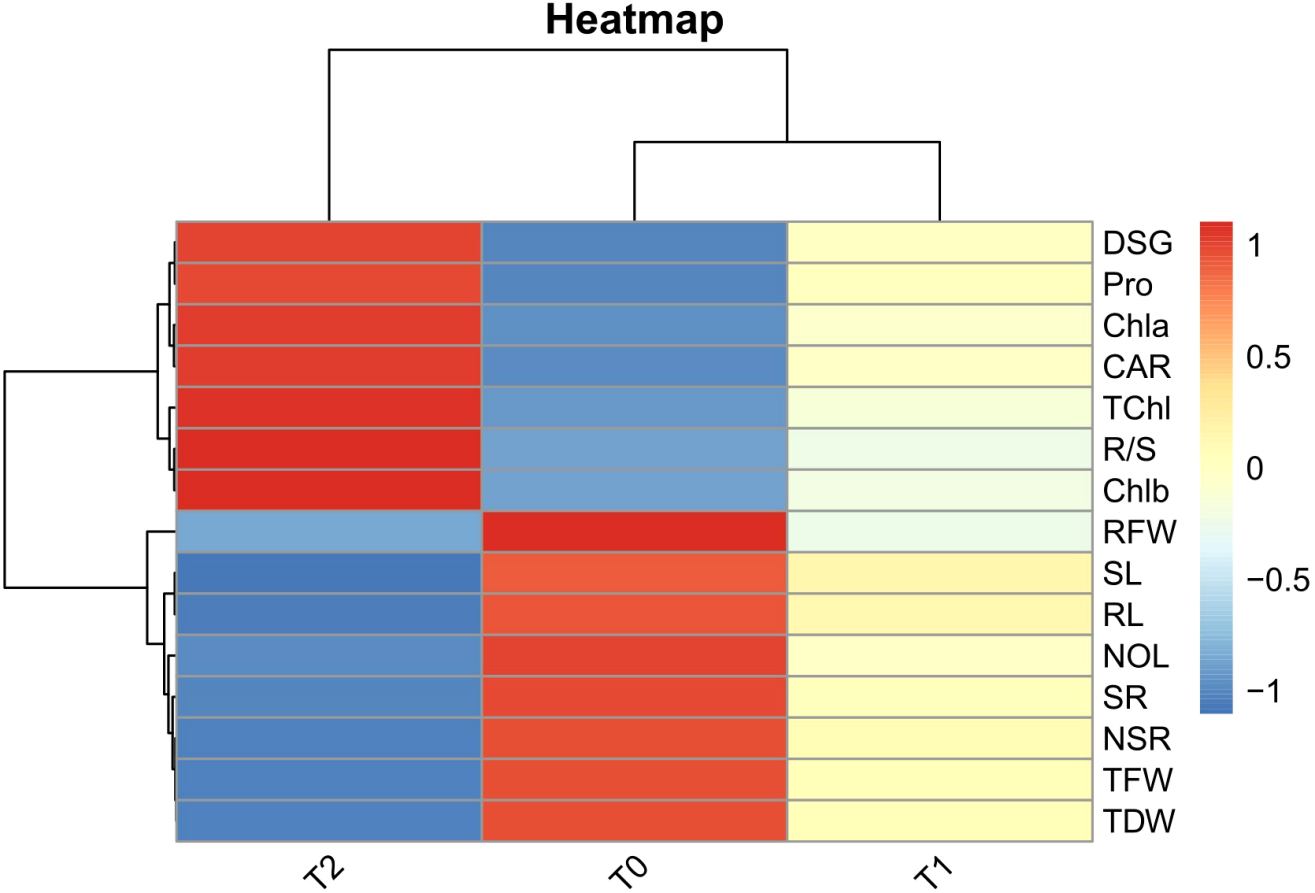
Heat maps of Pearson’s correlation coefficients among root architectural and biochemical parameters under three varying PEG 6000 concentrations as control (T0), 10% PEG-induced stress (T1), and 20% PEG-induced stress (T2). The colour scale on the right from darker red to darker blue represents the degree of correlation from high to low values across treatments for DSG (Days to seed germination), NOL (Number of leaves), SL (Shoot length), TFW (Total fresh weight), TDW (Total dry weight), SR (Survival rate), NSR (Number of secondary roots), RL (Root length), RFW (Root fresh weight), R/S (Root-to-shoot ratio).

The heatmap of Pearson correlation coefficients illustrates the relationships among growth, root, and biochemical traits under varying drought stress conditions (T0, T1, and T2) (**Figure 6**). Under control conditions (T0), growth traits (NOL, SL, TFW, TDW, SR) and root traits (NSR, RL, RFW) were strongly positively correlated. As drought stress intensified, these correlations decreased under T1 and became strongly negative under T2. In contrast, the root-to-shoot ratio (R/S) showed an opposite trend, shifting from a weak correlation at T0 to moderate under T1 and strong positive under T2. A similar pattern was observed for DSG, which progressed from a weak correlation at T0 to moderate and strong positive correlations under T1 and T2, respectively. Biochemical traits (Chla, Chlb, TChl, CAR, and Pro) also followed a reverse pattern compared to growth traits, with initially weak correlations under T0 that strengthened progressively under T1 and became strongly positive under T2.

### 3.3 Performance-based categorization of okra genotypes

#### 3.3.1 Score-based classification approach

The studied genotypes showed significant differences across different traits, including, DSG, NOL, TFW, TDW, SR, NSR, RL, SL, RFW, R/S, Chla, Chlb, TChl, CAR, and Pro, under three conditions (T0, T1, and T2), as indicated by their means and standard deviations (**Table 4**). Under controlled conditions (T0), genotype G10 achieved the highest score (38/45), followed by G6 (36/45), and G43 and G51 (35/45). In T1, G51 ranked highest (38/45), followed by G45 (37/45), with G10 and G6 each scoring 35/45. Under T2, G6 led with 37/45, followed by G23 and G51 (35/45), and G7, G45, and G54 (34/45). Conversely, the lowest scores under T0 were recorded for G5, G8, G49, and G55 (27/45). In T1, G55 had the lowest score (24/45), followed by G5 and G53 (25/45), while G8, G12, and G47 each scored 26/45. Under T2, G16 scored lowest (25/45), with G3, G12, and G47 close behind at 26/45. Considering overall performance across all conditions, G6 and G51 obtained the highest total scores (108/135), followed by G10 (106/135) and G45 (102/135). In contrast, G12, G47, and G55 had the lowest total scores (80/135), indicating poor drought tolerance.

**Table 4:** Categorization of studied genotypes into efficient (E), medium (M), and inefficient (I) types based on 15 estimated growth, root and biochemical parameters under three varying polyethylene glycol (PEG) concentrations in okra.

Genotypes	T0	Total (out of 45)	T1	Total (out of 45)	T2	Total (out of 45)	Overall (out of 135)
G1	M	29	M	31	M	31	91
G2	M	29	M	31	M	30	90
G3	M	31	M	28	I	26	85
G4	M	29	M	29	M	30	88
G5	I	27	I	25	M	30	82
G6	E	36	E	35	E	37	108
G7	M	31	M	33	E	34	98
G8	I	27	I	26	M	29	82
G9	M	32	M	29	E	34	95
G10	E	38	E	35	M	33	106
G11	M	30	M	31	M	28	89
G12	M	28	I	26	I	26	80
G13	M	30	M	32	M	31	93
G14	M	31	M	28	M	29	88
G15	M	28	M	31	M	28	87
G16	M	31	M	28	I	25	84
G17	M	28	M	29	M	29	86
G18	M	31	M	30	M	31	92
G19	M	28	M	28	M	33	89
G20	M	29	M	29	M	29	87
G21	M	30	M	27	M	30	87
G22	M	30	M	27	M	31	88
G23	M	28	M	33	E	35	96
G24	M	30	M	31	M	31	92
G25	M	29	M	30	M	29	88
G42	M	30	M	29	M	28	87
G43	E	35	M	30	M	29	94
G44	M	32	M	33	M	32	97
G45	M	31	E	37	E	34	102
G46	M	32	M	30	M	30	92
G47	M	28	I	26	I	26	80
G48	M	28	M	30	M	28	86
G49	I	27	M	30	M	31	88
G51	E	35	E	38	E	35	108
G53	M	29	I	25	M	29	83
G54	M	30	E	34	E	34	98
G55	I	27	I	24	M	29	80

#### 3.3.2 Index-based drought tolerance approach

**Figure 7:**
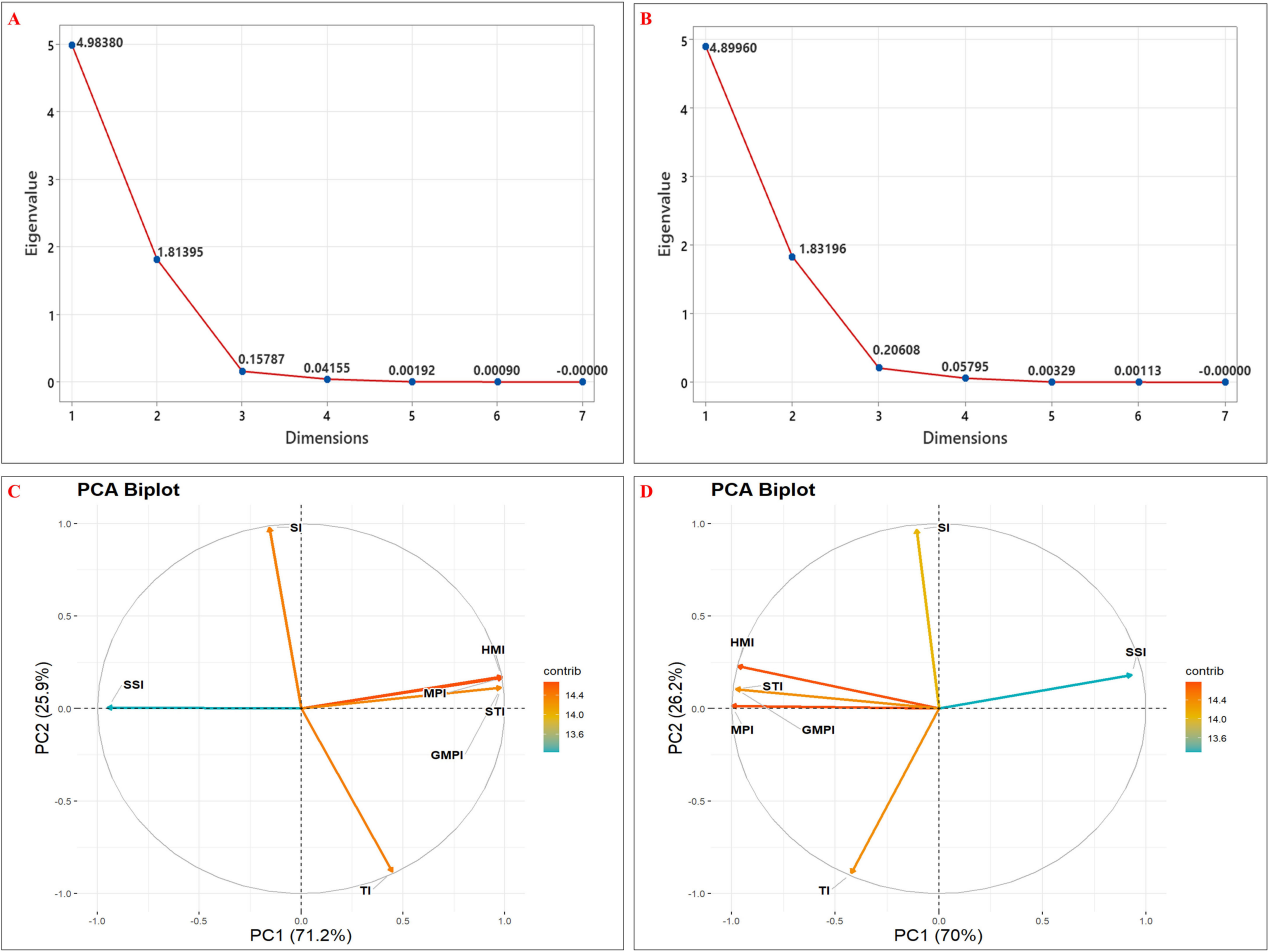
Principal component analysis (PCA) of stress tolerance indexes calculated for the total dry weight of studied genotypes. Scree plots of eigenvalues under **(A)** 10% and **(B)** 20% PEG 6000 stress respectively; PCA biplots showing variable contributions under **(C)** 10% and **(D)** 20% PEG stress respectively.

Stress Tolerance Score (STS) was calculated using seven stress tolerance indices for all genotypes (**Tables 5**, **6**). Among the evaluated genotypes, the highest STS was recorded for G45 (4.46), followed by G51 (4.11), while G49 (2.37) and G20 (2.59) had the lowest scores under T1 (**Table 5**). Similarly, in T2, G45 again had the highest STS (3.91), followed by G10 (3.19); the lowest values were noted for G20 (2.12) and G49 (1.88) (**Table 6**). To further identify which indices contributed the most to the variation, principal component analysis (PCA) was conducted using seven stress tolerance indices (SSI, MPI, GMPI, HMI, STI, TI and SI) for the evaluated genotypes. The scree plot results revealed that the first two components (PC1 and PC2) had eigenvalues greater than one under both stress levels (10% and 20%) (**Figures 7A, B**). In T1, PC1 and PC2 explained 71.20% and 25.91% of the total variation, respectively, with a cumulative variation of 97.11%. In T2, PC1 and PC2 accounted for 70% and 26.2% of the variance, respectively, resulting in a cumulative variation of 96.17%. The indices MPI and TI contributed most to PC1 and PC2, respectively (**Figures 7C, D**).

**Table 5:** Drought Stress tolerance indices of okra genotypes identified under 10% polyethylene glycol (PEG) concentration.

Genotypes	SSI	MPI	GMPI	HMI	STI	TI	SI	STS
G1	0.842	0.173	0.030	0.173	1.079	0.020	0.891	3.208
G2	0.874	0.170	0.029	0.170	1.040	0.013	0.925	3.220
G3	0.799	0.178	0.032	0.177	1.138	0.030	0.845	3.198
G4	0.663	0.202	0.040	0.198	1.437	0.057	0.754	3.350
G5	0.652	0.200	0.039	0.193	1.393	0.073	0.690	3.241
G6	0.682	0.203	0.041	0.202	1.480	0.033	0.848	3.490
G7	0.723	0.193	0.037	0.192	1.337	0.033	0.841	3.357
G8	1.051	0.140	0.019	0.139	0.700	0.027	0.826	2.902
G9	1.013	0.163	0.027	0.162	0.955	-0.027	1.178	3.471
G10	0.653	0.208	0.043	0.206	1.547	0.043	0.812	3.512
G11	0.721	0.195	0.038	0.194	1.362	0.030	0.857	3.397
G12	0.733	0.173	0.028	0.161	1.004	0.093	0.576	2.768
G13	0.775	0.180	0.032	0.178	1.153	0.040	0.800	3.158
G14	0.575	0.225	0.049	0.220	1.780	0.070	0.731	3.650
G15	0.679	0.192	0.035	0.184	1.271	0.077	0.667	3.105
G16	0.882	0.160	0.025	0.158	0.908	0.040	0.778	2.950
G17	0.952	0.147	0.021	0.143	0.756	0.047	0.725	2.790
G18	0.766	0.185	0.034	0.184	1.225	0.030	0.850	3.274
G19	0.618	0.207	0.041	0.198	1.472	0.087	0.653	3.274
G20	0.864	0.150	0.021	0.139	0.753	0.080	0.579	2.587
G21	1.206	0.122	0.015	0.120	0.525	0.030	0.780	2.798
G22	1.287	0.112	0.012	0.109	0.437	0.037	0.718	2.711
G23	0.994	0.142	0.020	0.138	0.706	0.043	0.735	2.778
G24	1.173	0.125	0.015	0.123	0.555	0.030	0.786	2.807
G25	0.702	0.192	0.036	0.187	1.295	0.057	0.742	3.211
G42	1.819	0.087	0.007	0.086	0.269	0.013	0.857	3.139
G43	1.680	0.108	0.012	0.107	0.418	-0.023	1.241	3.543
G44	1.487	0.100	0.010	0.098	0.354	0.027	0.765	2.841
G45	1.601	0.142	0.018	0.125	0.639	-0.097	2.036	4.464
G46	1.306	0.115	0.013	0.114	0.472	0.023	0.816	2.859
G47	1.832	0.083	0.007	0.082	0.247	0.020	0.786	3.057
G48	1.625	0.097	0.009	0.096	0.335	0.013	0.871	3.047
G49	0.810	0.150	0.019	0.129	0.695	0.113	0.452	2.368
G51	1.353	0.152	0.021	0.140	0.766	-0.083	1.758	4.107
G53	2.214	0.060	0.003	0.053	0.115	0.040	0.500	2.986
G54	1.686	0.107	0.011	0.106	0.406	-0.020	1.207	3.503
G55	2.694	0.047	0.002	0.038	0.064	0.040	0.400	3.285

SSI, stress susceptibility index; MPI, mean productivity index; GMPI, geometric mean productivity index; HMI, harmonic mean index; STI, stress tolerance index; TI, tolerance index; SI, stress index; STS, Stress tolerance score.

**Table 6:** Drought stress tolerance indices of okra genotypes identified under 20% polyethylene glycol (PEG) 6000 concentration.

Genotypes	SSI	MPI	GMPI	HMI	STI	TI	SI	STS
G1	0.859	0.165	0.027	0.163	0.969	0.037	0.800	3.019
G2	0.909	0.153	0.023	0.150	0.828	0.047	0.736	2.845
G3	0.824	0.165	0.026	0.160	0.952	0.057	0.707	2.891
G4	0.695	0.182	0.031	0.169	1.105	0.097	0.580	2.858
G5	0.668	0.190	0.034	0.179	1.223	0.093	0.606	2.992
G6	0.713	0.185	0.033	0.178	1.189	0.070	0.682	3.050
G7	0.756	0.175	0.029	0.168	1.060	0.070	0.667	2.924
G8	1.079	0.128	0.016	0.123	0.571	0.050	0.674	2.642
G9	1.078	0.137	0.019	0.135	0.667	0.027	0.822	2.885
G10	0.674	0.195	0.037	0.189	1.326	0.070	0.696	3.186
G11	0.773	0.165	0.025	0.153	0.908	0.090	0.571	2.686
G12	0.772	0.150	0.018	0.117	0.634	0.140	0.364	2.195
G13	0.803	0.165	0.026	0.158	0.937	0.070	0.650	2.808
G14	0.610	0.200	0.036	0.182	1.312	0.120	0.538	2.999
G15	0.730	0.160	0.021	0.129	0.746	0.140	0.391	2.317
G16	0.919	0.142	0.019	0.131	0.670	0.077	0.574	2.532
G17	0.999	0.125	0.014	0.109	0.490	0.090	0.471	2.297
G18	0.812	0.160	0.024	0.150	0.865	0.080	0.600	2.691
G19	0.679	0.165	0.020	0.121	0.721	0.170	0.320	2.196
G20	0.903	0.130	0.013	0.102	0.479	0.120	0.368	2.117
G21	1.242	0.108	0.011	0.101	0.394	0.057	0.585	2.498
G22	1.348	0.090	0.007	0.072	0.234	0.080	0.385	2.216
G23	1.028	0.127	0.015	0.116	0.530	0.073	0.551	2.440
G24	1.213	0.110	0.011	0.102	0.404	0.060	0.571	2.471
G25	0.738	0.170	0.026	0.155	0.951	0.100	0.545	2.687
G42	1.832	0.083	0.007	0.082	0.247	0.020	0.786	3.057
G43	1.737	0.093	0.009	0.093	0.314	0.007	0.931	3.184
G44	1.531	0.087	0.007	0.078	0.245	0.053	0.529	2.530
G45	1.694	0.118	0.013	0.113	0.482	-0.050	1.536	3.906
G46	1.340	0.103	0.010	0.098	0.365	0.047	0.632	2.595
G47	1.918	0.062	0.003	0.045	0.101	0.063	0.321	2.513
G48	1.714	0.072	0.004	0.058	0.149	0.063	0.387	2.447
G49	0.845	0.130	0.011	0.085	0.397	0.153	0.258	1.880
G51	1.504	0.107	0.011	0.107	0.410	0.007	0.939	3.085
G53	2.253	0.052	0.002	0.036	0.067	0.057	0.292	2.758
G54	1.743	0.092	0.008	0.091	0.302	0.010	0.897	3.143
G55	2.722	0.042	0.001	0.027	0.040	0.050	0.250	3.131

SSI, stress susceptibility index; MPI, mean productivity index; GMPI, geometric mean productivity index; HMI, harmonic mean index; STI, stress tolerance index; TI, tolerance index; SI, stress index; STS, Stress tolerance score.

The classification of genotypes into susceptible, moderately tolerant, and tolerant groups were done through comprehensive strategy combining the Score-Based Classification approach and the Index-Based Drought Tolerance Approach. Genotypes such as G45 and G51 demonstrated consistently high performance in both approaches and trait specific responses, so were classified as tolerant. G47 showed a moderate performance in trait values and STS, was categorized as moderately tolerant, while G19 and G49 with low performance in root traits, with comparatively low scores and index values, were identified as susceptible. The physiological validity of genotype classification was further supported by oxidative stress markers. Genotypes like G45 and G6, identified as drought-tolerant based on scoring and indices, also showed the lowest MDA and H_2_O_2_ accumulation under T2, indicating minimal oxidative damage. In contrast, G19, classified as susceptible, exhibited the highest levels of these markers, further confirming its sensitivity to drought.

### 3.4 Field emission scanning electron microscopy

**Figure 8:**
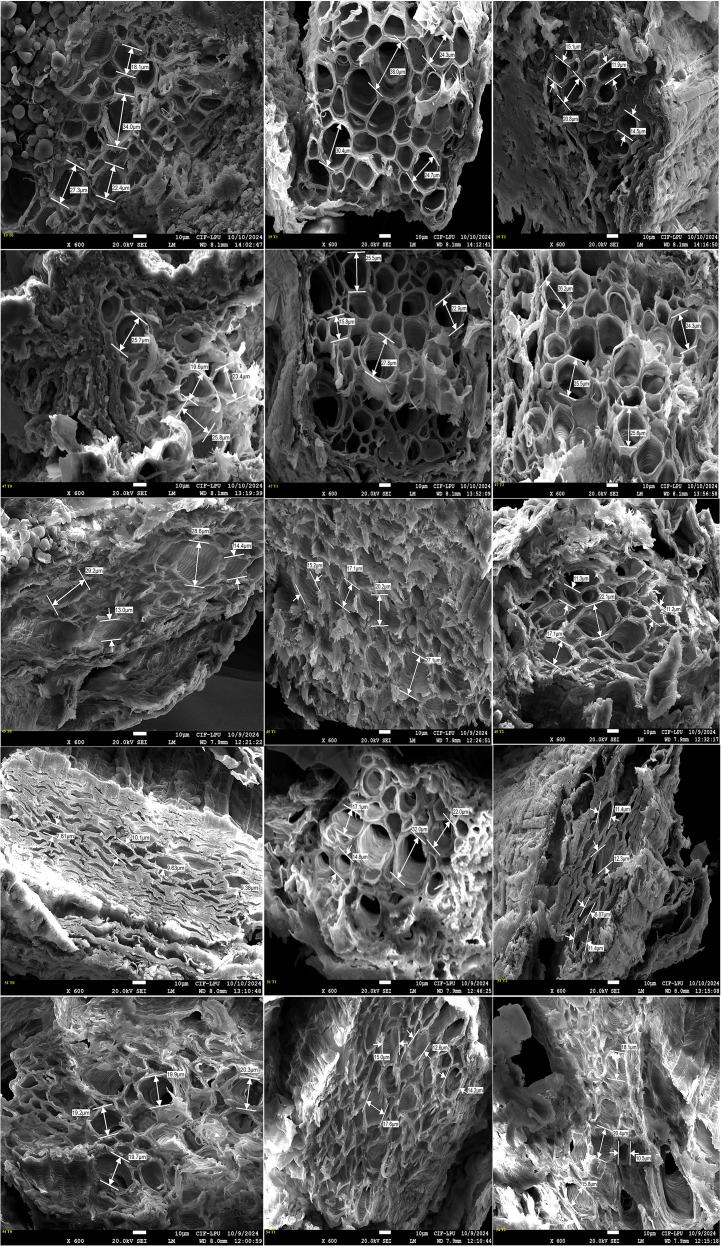
Field Emission Scanning Electron Microscopy (FESEM) images illustrating cross sectional areas of primary root xylem vessel at magnification of x 600 and scale bar 10 µm across five okra genotypes: susceptible (19), moderately tolerant (47), tolerant (45 and 51), and check (54) under three PEG concentrations: control (T0), 10% (T1), and 20% (T2). The image shows genotypic specific structural modifications in response to drought stress, with susceptible genotype displaying reduced vessel diameter at higher stress level, while tolerant genotypes maintain stable xylem structure by adapting to the stress conditions.

**Figure 9:**
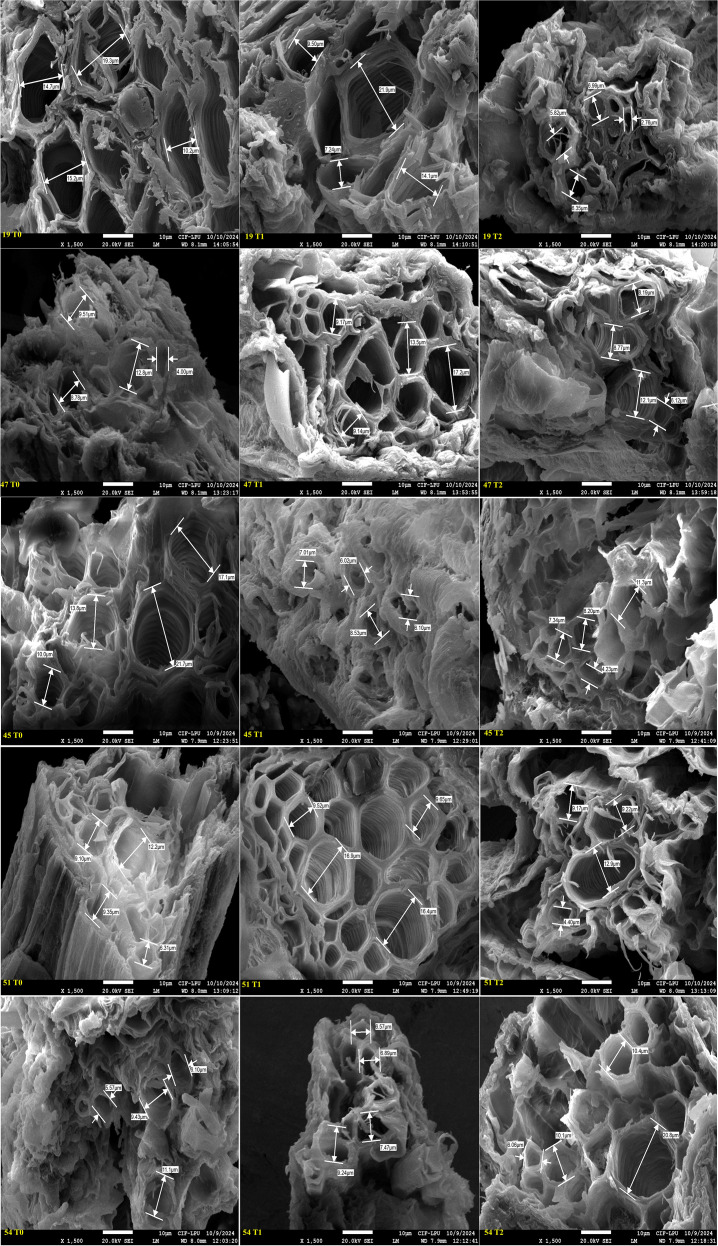
Field Emission Scanning Electron microscopy (FESEM) images illustrating cross sectional areas of secondary root xylem vessel at magnification of x 1500 and scale bar 10 µm across five okra genotypes: susceptible (19), moderately tolerant (47), tolerant (45 and 51), and check (54) under three PEG concentrations: control (T0), 10% (T1), and 20% (T2). Genotypes exhibited distinct anatomical responses to drought stress, with susceptible genotype showing progressive vessel diameter reduction, while other genotypes maintaining structural stability in response to increasing stress indicating an adaptative response.

**Figure 10:**
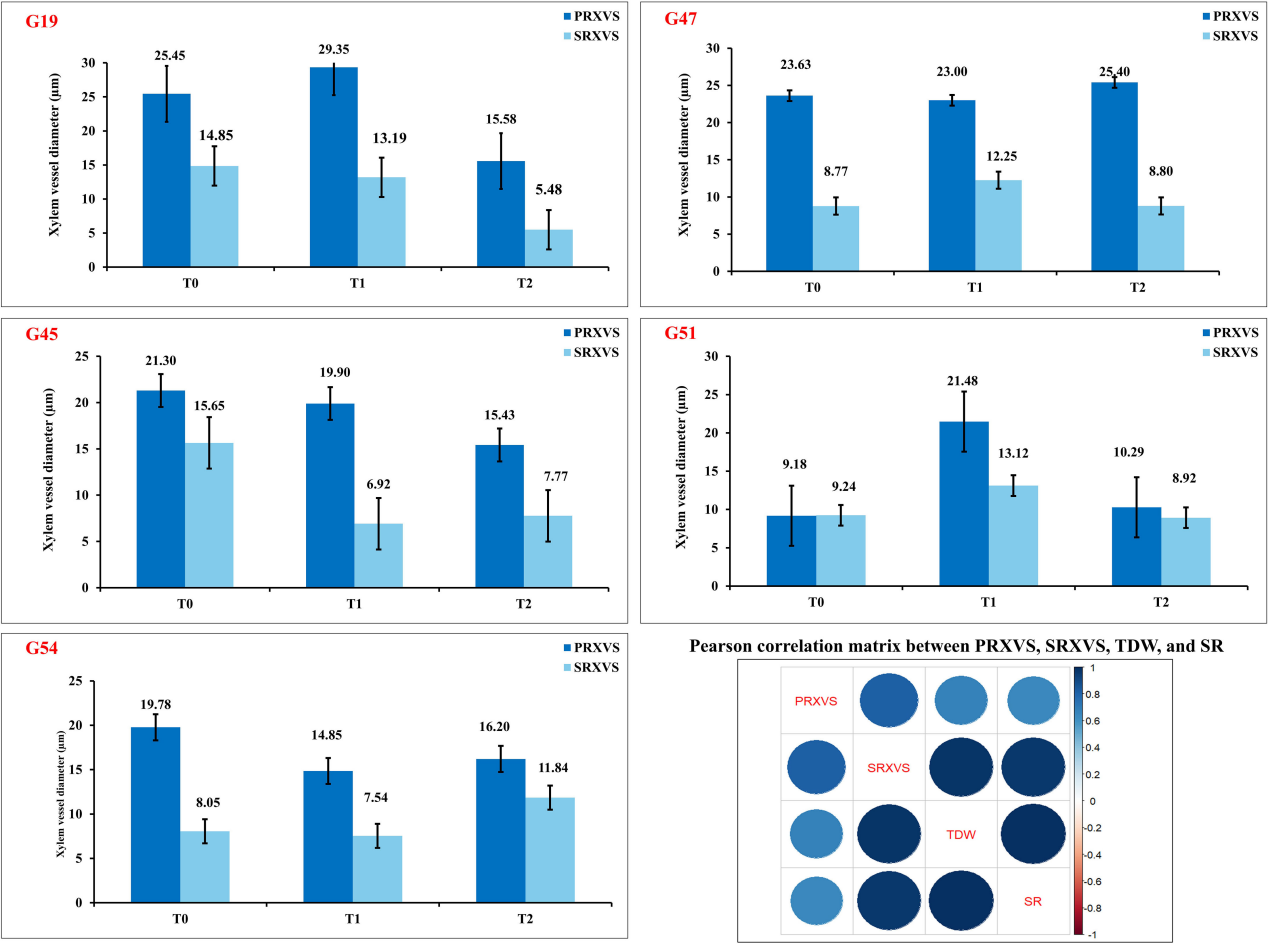
Variation in root xylem vessel diameter across drought stress treatments in okra genotypes classified as susceptible (G19), moderately tolerant (G47), tolerant (G45 and G51), and check (G54). Bar plots represent primary root xylem vessel size (PRXVS, dark blue) and secondary root xylem vessel size (SRXVS, light blue) under three PEG-induced stress levels: control (T0), moderate (T1), and severe (T2). Error bars denote standard error of the mean. Correlation matrix depicts relationships among PRXVS, SRXVS, total dry weight (TDW), and survival rate (SR), with circle size and color (dark blue = strong positive) reflecting correlation strength and direction.

Genotypes representing susceptible, moderately tolerant, tolerant, and check categories were further validated for their PRXVS (**Figure 8**) and SRXVS (**Figure 9**) using FESEM under three treatment conditions. The susceptible genotype G19 showed a 15.32% increase in PRXVS under T1, followed by a 38.80% decrease under T2 compared to T0. In SRXVS, reductions of 11.21% in T1 and 63.09% in T2 were recorded. The moderately tolerant genotype G47 exhibited a slight decrease in PRXVS (2.64%) under T1, followed by a 7.51% increase in T2. SRXVS, on the other hand, increased markedly by 39.66% in T1 and marginally by 0.25% in T2. In the tolerant genotype G45, PRXVS declined by 6.57% in T1 and 27.58% in T2, while SRXVS showed a substantial reduction of 55.81% in T1 and 50.36% in T2. For another tolerant genotype, G51, PRXVS increased slightly by 3.55% in T1 and decreased by 0.59% in T2. SRXVS increased by 41.96% in T1 and then declined by 3.43% in T2. The check genotype G54 showed a decrease in PRXVS by 4.50% in T1 and 9.12% in T2. In contrast, SRXVS decreased by 6.30% in T1 but increased significantly by 47.80% in T2 (**Figure 10**). These observations indicate genotype-specific responses in xylem vessel dimensions under water deficit conditions, reflecting diverse drought tolerance mechanisms. To further understand the anatomical basis of drought tolerance, a correlation analysis was conducted between PRXVS, SRXVS, TDW, and SR (**Figure 10**). Both PRXVS and SRXVS showed strong positive correlations with TDW and SR, indicating the role of xylem modifications in plant survival and biomass accumulation under osmotic stress.

## 4 Discussion

**Figure 11:**
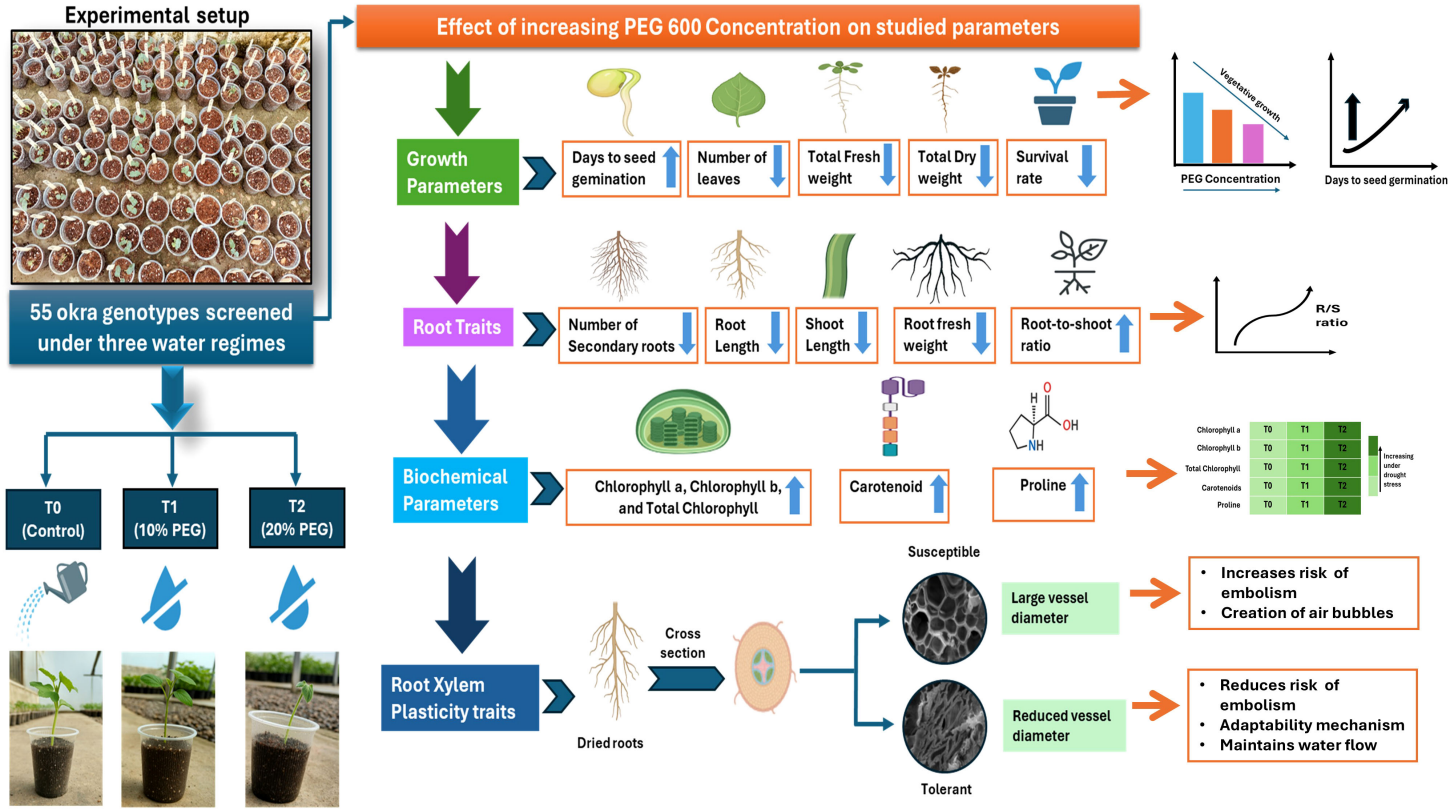
Schematic representation of experimental setup for screening 55 okra genotypes at seedling stage under three polyethylene glycol (PEG) 600 regimes: control (T0, without PEG)), 10% PEG (T1), 20% PEG (T2). Figure shows the effect of increasing PEG induced drought stress on growth, root, and biochemical parameters, and root xylem plasticity.

Water deficit is a major abiotic stress that adversely affects okra productivity by disrupting key morpho-physiological processes such as plant growth, photosynthesis, and chlorophyll synthesis (Razi et al., 2021). While drought tolerance varies both across crops and among genotypes within a species (Udpuay et al., 2024), this study focused on assessing the effects of varying drought stress levels on root plasticity and physio-biochemical traits in okra (**Figure 11**). ANOVA revealed significant effects of genotype, treatment, and their interaction, indicating substantial genetic variability. Tukey’s test further confirmed the significant impact of drought on growth, root, and biochemical traits among studied genotypes. Drought stress notably reduced biomass and morphological parameters, along with seed germination, likely due to disrupted metabolism (Saha et al., 2022). Delayed germination under stress may also serve as a survival mechanism (Guo M. et al., 2024).

Growth-related parameters such as NOL, SL, TFW, TDW, and SR were all significantly reduced under drought stress. These reductions can be attributed to decreased water content, which lowers turgor pressure and water potential, leading to stomatal closure, inhibited cell elongation, and ultimately reduced biomass accumulation (Zia et al., 2021). Among the root traits, NSR, RL, and RFW declined significantly under drought, while the R/S increased. This shift suggests a strategic allocation of resources toward root development, enabling plants to access deeper soil moisture. A similar trend was observed by Bukan et al. (2024) in soybean, where root traits declined under drought, yet the R/S ratio increased as plants prioritized water uptake. Metabolic adjustments under stress likely promote root growth over shoot growth, thereby sustaining root length despite significant shoot reduction (Kalra et al., 2024).

Chlorophyll content, typically reduced under drought due to oxidative damage (Ansari et al., 2019), showed a different trend in our study. We observed an increase in chlorophyll a, b, and total chlorophyll under drought stress, suggesting an adaptive response wherein pigment production is enhanced to maintain photosynthetic activity. This aligns with findings in *Cyamopsis tetragonoloba*, where chlorophyll levels were elevated during early drought stages but declined later (Shanthi et al., 2023). The maintenance of chlorophyll may also be supported by the accumulation of osmolytes and antioxidants, which protect against ROS and help stabilize photosynthetic pigments (Yang et al., 2021). Increased carotenoid levels, observed in our study, further indicate photoprotective adaptation under drought. Carotenoids safeguard photosynthetic machinery from oxidative damage and have been reported to increase under drought in various plant species (Sherin et al., 2022; Kuru et al., 2021; Cai et al., 2005; Yang et al., 2015). Additionally, we recorded a significant rise in proline content under drought conditions. Proline functions as a vital osmoprotectant and ROS scavenger, contributing to cellular water balance, protein stabilization, and oxidative stress mitigation (Hosseinifard et al., 2022). The elevated proline levels in the T2 treatment indicate a strong biochemical defense response, underscoring its clearrole in drought tolerance mechanisms.

While the majority of traits in this study were evaluated across all three PEG-induced regimes (T0, T1, and T2), MDA and H_2_O_2_ measurements were restricted to T0 and T2 to focus on the oxidative stress extremes under control and severe drought conditions. This approach allowed for a high-resolution contrast, effectively capturing drought-induced lipid peroxidation and ROS accumulation without redundancy (Farooq et al., 2010; Mihaljevic et al., 2021). Despite being limited to two treatments, the trends observed in MDA and H_2_O_2_ levels strongly aligned with reductions in biomass, survival rate, and root traits under T2, supporting their relevance as physiological markers of drought damage. Notably, genotypes like G6 and G45 exhibited lower MDA and H_2_O_2_ levels under T2, consistent with their superior performance in growth, survival, and xylem plasticity. This coherence underscores the potential of integrating oxidative stress markers into drought tolerance screening, particularly as a validation layer for phenotypic classifications derived from morphological and anatomical traits.

### 4.1 Drought stress delays germination and triggers adaptive responses

Drought stress disrupts metabolic processes, delaying seed germination and establishment. Mustamu et al. (2023) reported a 49.14% decrease in germination speed with a 50% increase in PEG concentration. Consistent with this finding, our study revealed that DSG increased significantly with higher concentrations of PEG6000, indicating that drought prolongs the germination process. Furthermore, correlation analysis demonstrated that as drought intensity escalated, the normally positive correlations among growth and root traits weakened. This decline in the coordinated response among traits under water scarcity aligns with the observations of Alshammari et al. (2024) and is further supported by the studies of Rida et al. (2021); Zhao et al. (2021), and Verma and Sarma (2021), all of which underscore the profound impact of limited water availability on plant development.

Biomass allocation also shifted under drought stress. Our findings showed that the R/Sratio increased with higher PEG6000 concentrations, suggesting that shoot growth was more adversely affected than root growth. This adaptive response, aimed at enhancing water uptake by favoring root development, is in agreement with Beyaz and Uslu (2025) and highlights the critical role of the R/S ratio in plant stress adaptation (Tavares et al., 2021). Biochemical parameters, which are essential for mitigating oxidative damage and maintaining physiological functions, also responded to drought. Under control conditions, these parameters were weakly correlated; however, under mild drought stress (T1), a moderate positive correlation emerged, and under severe drought stress (T2), the correlation became strong. This trend is in line with the findings of Shanthi et al. (2023), who reported increases in chlorophyll and carotenoid levels during the early stages of drought stress. Together, these results illustrate how drought stress not only delays germination but also triggers adaptive changes in growth, biomass allocation, and biochemical defense mechanisms, reflecting a multifaceted plant response to water scarcity.

### 4.2 Integrative assessment of phenotypic traits and xylem adaptations enables effective selection of drought-tolerant okra genotypes

The success of a breeding program depends on selecting genotypes that perform well under both normal and stressful conditions. Previous research has employed various selection criteria, for instance, standard deviation‐based methods, biplot‐based classification, bivariate classification methods, and stress tolerance index‐based classification, in wheat, mungbean, and brassica, respectively, for genotype classification (Gill et al., 2004; Kosar et al., 2003; Gunes et al., 2006). In the present study, genotypes were classified as efficient, medium, or inefficient based on cumulative scores derived from growth, root, and biochemical traits under three conditions (T0, T1, T2), following a scoring approach adopted from Reddy et al. (2021). Similar methodologies applied in wheat and Brassica (Bilal et al., 2018; Aziz et al., 2011) emphasize that superior performance under control conditions may not necessarily indicate stress tolerance (Manske et al., 2000; Hammond and White, 2008). Genotypes G6 and G51 showed the highest drought tolerance, followed by G10 and G45, attributed to strong root traits and biomass. In contrast, G12, G47, and G55 had the lowest scores due to poor root development and dry weight.

Thiry et al. (2016) and Grzesiak et al. (2019) classified genotypes using stress tolerance indices based on total dry weight under control and stress conditions. A similar approach was applied in this study to assess genotype adaptability, as it allows for the identification of genotypes that maintain higher biomass production despite stress. The indices SSI, TI, and SI are susceptibility indicators that show a negative correlation with biomass and effectively distinguish between drought-tolerant and susceptible genotypes (Sareen et al., 2012), while MPI, GMPI, HMI, and STI are tolerance indices positively associated with biomass, thereby identifying genotypes with both high biomass production and stress tolerance (Khodarahmpour et al., 2011). In the present study, PCA analysis indicated that two indices, MPI and TI, explained the greatest percentage of variation among the studied indices. Although individual tolerance and susceptibility indices alone may fail to identify stress-tolerant genotypes with high biomass under both control and stress conditions (Khayatnezhad et al., 2010), our findings, as suggested by Thiry et al. (2016), highlight that integrating both sets of indices offers a robust approach for identifying stable, drought-tolerant genotypes. In our study, genotype G45 showed the highest STC score at both levels of stress (T1 and T2), indicating its high efficiency under varied drought stress conditions.

FESEM is a powerful imaging technique that provides high-resolution visualization of cellular structures, allowing detailed analysis of xylem vessel morphology. Previous studies have used this technique to investigate root plasticity under drought conditions in maize (Sandhu et al., 2023) and wheat (Licaj et al., 2023). In the present study, FESEM analysis revealed significant variations in primary and secondary xylem vessel diameter among genotypes, highlighting the impact of stress responses on vascular development. For example, the susceptible genotype G19 initially showed increased PRXVS, likely due to delayed stress perception, which led to larger vessels vulnerable to embolism. In contrast, as drought stress intensified, PRXVS shrank, limiting growth and leading to mortality, while SRXVS decreased progressively, indicating an earlier defensive response. This is in line with findings that secondary root tracheid diameters decline significantly under prolonged drought across various stress levels (Li et al., 2024), highlighting distinct adaptation strategies for primary and secondary roots in drought resilience (Irshad et al., 2024; Ranjan et al., 2022). The moderately tolerant genotype G47 initially decreased PRXVS, likely as an adaptive strategy to minimize embolism risk under mild drought; however, under extreme stress, PRXVS increased to maximize water flow even at the risk of embolism (Flor et al., 2025). In secondary roots, xylem vessels expanded to access moisture from deeper soil layers as a key drought mechanism (Kumar et al., 2017). Under mild stress, PRXVS decreased to prevent embolism, while SRXVS expanded for better water uptake. Under severe stress, PRXVS increased to maintain flow despite the risk, whereas SRXVS stabilized, indicating a shift in drought adaptation.

The tolerant genotype G45 reduced PRXVS under mild drought to minimize embolism risk, consistent with the idea that smaller vessels enhance drought resistance (Olson, 2023). As drought stress increased, PRXVS further reduced to protect the vascular system from permanent damage (Qaderi et al., 2019). The study aligns with findings that narrower xylem vessels are advantageous under drought, while larger vessels promote growth in water-abundant conditions (Priatama et al., 2022). SRXVS declined sharply, suggesting a hydraulic segmentation strategy where water flow in secondary roots is restricted to safeguard the primary root system. This strategy prioritizes deeper water access through primary roots while limiting water loss from secondary roots in the surface soil (Kang et al., 2022).

The tolerant genotype G51 showed a moderate decrease in PRXVS under mild stress to balance water conductivity with embolism risk (Lovisolo and Schubert, 1998; Haworth et al., 2018; Dolezal et al., 2019), while maintaining stability in severe drought, thereby preserving hydraulic conductivity. SRXVS initially increased, indicating enhanced surface water uptake, but later declined under severe drought, suggesting that primary roots prioritize long-term stability while secondary roots adjust dynamically to water availability (Shoaib et al., 2022). The check genotype G54 exhibited a decrease in PRXVS in T1 to conserve water and maintain stable flow, with a further reduction in T2 signifying an adaptive response to prolonged drought. Initially, SRXVS decreased in T1 to limit water loss under mild stress, but in T2, SRXVS sharply increased, suggesting a delayed compensation mechanism where secondary roots become active for water uptake under severe drought. This response aligns with findings that drought stress modulates hormonal interactions, resulting in initial xylem restriction followed by increased differentiation in secondary roots as drought stress persists (Jang and Choi, 2018). The correlation among root xylem vessel size, dry biomass accumulation, and survival rate revealed a strong positive association between root anatomical traits and key indicators of drought tolerance (TDW and SR) under stress conditions. This highlights the critical role of root xylem vessel plasticity in enhancing both plant survival and productivity under limited water availability (Prince et al., 2017).

In summary, this study demonstrates that an integrative approach, combining cumulative performance scoring across multiple growth and biochemical traits with stress tolerance indices and FESEM-based vascular analysis, offers a robust framework for identifying and breeding drought-tolerant okra genotypes. While this study provides valuable insights into the morphological, anatomical,and biochemical mechanisms underlying drought tolerance in okra, future research should expand on these findings by incorporating root metabolic profiling. Specifically, analyzing the dynamics of sugars, organic acids, and other key metabolites will offer a more comprehensive understanding of root plasticity and its role in drought adaptation. Integrating these metabolic traits with anatomical and physiological data will not only deepen our mechanistic understanding but also enhance the precision of breeding strategies aimed at developing resilient okra cultivars for water-limited environments. Furthermore, the contrasting genotypes identified in this study offer a strong foundation for developing trait-focused mapping populations to dissect the genetic basis of drought resilience. Crosses between tolerant (e.g., G6, G51) and susceptible (e.g., G12, G47) lines can yield segregating progenies for mapping QTLs related to root plasticity, antioxidant defense, and biomass stability under stress. Their inclusion in GWAS panels will improve allelic diversity and mapping resolution, aiding the identification of candidate genes and markers for selection. Additionally, these genotypes can be used as training sets in genomic prediction models to accelerate breeding of drought-tolerant okra cultivars.

## 5 Conclusion

Current study highlights the critical role of root plasticity, xylem traits, and biochemical responses in enhancing drought tolerance in okra. Based on overall performance, genotypes G51 (Sonam), G6 (HAU-480), G10 (Bhindi Champion), and G45 (Pooja-01) emerged as the most drought-tolerant, exhibiting superior root development and biomass accumulation under PEG-induced stress condition. Increased root-to-shoot ratio and proline accumulation reflected adaptive responses. PCA emphasized the importance of mean productivity and tolerance indices in selection. These insights provide a strong basis for breeding drought-resilient okra suited to arid and semi-arid regions.

## Data Availability

The raw data supporting the conclusions of this article will be made available by the authors, without undue reservation.

## Supplementary material

Supplementary Figure 1 Genotypic variation in growth parameters of okra under three levels of polyethylene glycol (PEG)-induced drought stress: control (T0, without PEG), moderate stress (T1), and severe stress (T2). Line plots represent treatment-wise mean values of **(A)** Days to Seed Germination (DSG), **(C)** Number of Leaves (NOL), **(E)** Total Fresh Weight (TFW), **(G)** Total Dry Weight (TDW), and **(I)** Survival Rate (SR) across 55 genotypes. Values above each point indicate trait means for individual genotypes. Corresponding regression plots **(B, D, F, H, J)** illustrate linear relationships among treatment levels with corresponding R² values.

Supplementary Figure 2 Genotypic variation in root traits of okra under three levels of polyethylene glycol (PEG)-induced drought stress: control (T0), moderate stress (T1), and severe stress (T2). Line plots represent treatment-wise mean values of **(A)** Number of secondary roots (NSR), **(C)** Root length (RL), **(E)** Shoot length (SL), **(G)** Root fresh weight (RFW), **(I)** Root-to-shoot-ratio (R/S) across 55 genotypes. Values above each point indicate trait means for individual genotypes. Corresponding regression plots **(B, D, F, H, J)** illustrate linear relationships among treatment levels with corresponding R² values.

Supplementary Figure 3 Genotypic variation in biochemical parameters of okra under three levels of polyethylene glycol (PEG)-induced drought stress: control (T0), moderate stress (T1), and severe stress (T2). Line plots represent treatment-wise mean values of **(A)** Chlorophyll a (Chla), **(C)** Chlorophyll b (Chlb), **(E)** Total Chlorophyll (TChl), **(G)** Carotenoid (CAR), **(I)** Proline (Pro) across 55 genotypes. Values above each point indicate trait means for individual genotypes. Corresponding regression plots **(B, D, F, H, J)** illustrate linear relationships among treatment levels with corresponding R² values.

Supplementary Figure 4 Genotypic variation in **(A)** Malondialdehyde (MDA), **(C)** Hydrogen peroxide (H_2_O_2_) under control (T0) and severe stress (T2) induced by different levels of polyethylene glycol (PEG) 6000. Line plots represent treatment-wise mean values across 55 genotypes. Values above each point indicate trait means for individual genotypes. Corresponding regression plots **(B, D)** illustrate linear relationships among treatment levels with corresponding R² values.
