# Supplementary figures and images for: Root plasticity and xylem modifications drive drought resilience in okra [Abelmoschus esculentus (L.) Moench] at the seedling stage
Source: Front Plant Sci. 2025 Nov 19;16:1630935. doi: 10.3389/fpls.2025.1630935 (PMC12672279; doi:10.3389/fpls.2025.1630935)

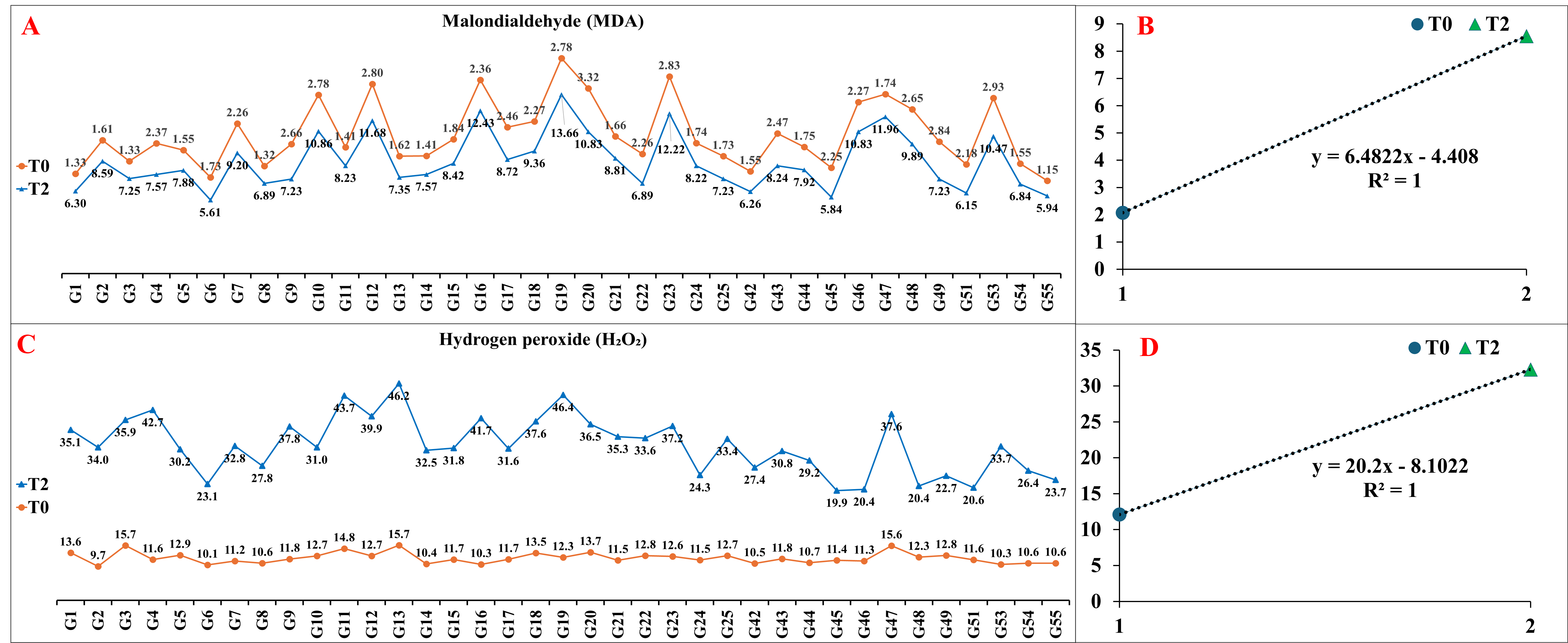

Supplement: Supplementary Figure 4 — Genotypic variation in (A) Malondialdehyde (MDA), (C) Hydrogen peroxide (H2O2) under control (T0) and severe stress (T2) induced by different levels of polyethylene glycol (PEG) 6000. Line plots represent treatment-wise mean values across 55 genotypes. Values above each point indicate trait means for individual genotypes. Corresponding regression plots (B, D) illustrate linear relationships among treatment levels with corresponding R² values. [file Image4.tiff]
