# Supplementary material for: Root plasticity and xylem modifications drive drought resilience in okra [Abelmoschus esculentus (L.) Moench] at the seedling stage
Source: Front Plant Sci. 2025 Nov 19;16:1630935. doi: 10.3389/fpls.2025.1630935 (PMC12672279; doi:10.3389/fpls.2025.1630935)
Supplement: Supplementary file 5 [file Table1.docx]

| **Supplementary Table 1: List of studied Okra (*Abelmoschus esculentus* L.) genotypes** | | |
| --- | --- | --- |
| **Genotypes** | **Name** | **Source** |
| **G1** | MS-1031 | Mahavira Seed Corporation |
| **G2** | Hari Kranti | IIVR, Varanasi |
| **G3** | Bhindi Panchwati | Doctor Seeds |
| **G4** | Jing orange | Earthjoy Farm |
| **G5** | Clemson Spinless | Biosnyg |
| **G6** | HAU-480 | HAU |
| **G7** | Perkins long okra | Biosnyg |
| **G8** | Star of David | Biosnyg |
| **G9** | Bhindi S-51 | India Seeds Syndicate |
| **G10** | Bhindi champion RK | India Seeds Syndicate |
| **G11** | Maharani | Narindra Seeds |
| **G12** | Arka Anamika | IARI, New Delhi |
| **G13** | Arka Rg Rani | IARI, New Delhi |
| **G14** | Modern | Biosnyg |
| **G15** | Prabhani Kranti | VNMK, Prabhani |
| **G16** | Phule Vimukta | MPKV, Rahuri |
| **G17** | Imp Glory | Jainson Agri Store |
| **G18** | Bhindi sonal gold | India Seeds Syndicate |
| **G19** | Punjab No.13 | PAU, Ludhiana |
| **G20** | Hina RCH | Calyx Seeds |
| **G21** | Selection -040 | India Seeds Syndicate |
| **G22** | Mahyco B-L-101 | Mahyco |
| **G23** | Bhindi Bhagyashree | India Seeds Syndicate |
| **G24** | Ksp 188 | India Seeds Syndicate |
| **G25** | Kashi Pragati | IARI, New Delhi |
| **G26** | GFS Gold | Gujarat Farm Seeds |
| **G27** | IC052299 | NBPGR, New Delhi |
| **G28** | IC052301 | NBPGR, New Delhi |
| **G29** | IC052302 | NBPGR, New Delhi |
| **G30** | IC052303 | NBPGR, New Delhi |
| **G31** | IC052312 | NBPGR, New Delhi |
| **G32** | IC052321 | NBPGR, New Delhi |
| **G33** | IC052322 | NBPGR, New Delhi |
| **G34** | IC057733 | NBPGR, New Delhi |
| **G35** | IC058235 | NBPGR, New Delhi |
| **G36** | IC058704 | NBPGR, New Delhi |
| **G37** | IC058710 | NBPGR, New Delhi |
| **G38** | IC058712 | NBPGR, New Delhi |
| **G39** | IC058768 | NBPGR, New Delhi |
| **G40** | IC086008 | NBPGR, New Delhi |
| **G41** | IC089712 | NBPGR, New Delhi |
| **G42** | Agri Anamika | Imperial Agro Genetics |
| **G43** | Heena | Manvik Seeds |
| **G44** | Bhindi imperial bahar | Imperial Agro Genetics |
| **G45** | Pooja-01 | Agriculture India Seeds |
| **G46** | Red Burgandy | Biosnyg |
| **G47** | High cut | PAU, Ludhiana |
| **G48** | Okra -3030 | Doctor Seeds |
| **G49** | Khushi | Kaveri Seeds |
| **G50** | Emerald green | PAU, Ludhiana |
| **G51** | Sonam | Namdhari seeds |
| **G52** | Somya | Doctor Seeds |
| **G53** | IARI 728 | IARI, New Delhi |
| **G54** | Pusa Sawani | IARI, New Delhi |
| **G55** | Hari Rani | Jai Bhawani seeds and Pesticides |

| **Supplementary Table 2: List of studied traits abbreviations** | | | |
| --- | --- | --- | --- |
| **Classification** | **Trait description** | **Abbreviations** | **Units** |
| Growth Parameters (GP) | Days to seed germination | DSG | Days |
|  | Number of leaves | NOL | Count |
|  | Total fresh weight | TFW | g |
|  | Total dry weight | TDW | g |
|  | Survival rate | SR | % |
| Root Traits | Number of secondary roots | NSR | Count |
|  | Root length | RL | cm |
|  | Shoot length | SL | cm |
|  | Root fresh weight | RFW | g |
|  | Root-to-shoot ratio | R/S | --- |
| Biochemical Parameters (BP) | Chlorophyll a | Chla | mg/g |
|  | Chlorophyll b | Chlb | mg/g |
|  | Total Chlorophyll | TChl | mg/g |
|  | Carotenoid | CAR | mg/g |
|  | Proline | Pro | µmol/g |
| Root Xylem Plasticity traits | Primary root xylem vessel size | PRXVS | µm |
|  | Secondary root xylem vessel size | SRXVS | µm |
